# Redundant neural circuits regulate olfactory integration

**DOI:** 10.1371/journal.pgen.1010029

**Published:** 2022-01-31

**Authors:** Wenxing Yang, Taihong Wu, Shasha Tu, Yuang Qin, Chengchen Shen, Jiangyun Li, Myung-Kyu Choi, Fengyun Duan, Yun Zhang

**Affiliations:** 1 Department of Organismic and Evolutionary Biology, Center for Brain Science, Harvard University, Cambridge, Massachusetts, United States of America; 2 Department of Physiology, West China School of Basic Medical Sciences and Forensic Medicine, Sichuan University, Chengdu, Sichuan, China; Northeastern University, UNITED STATES

## Abstract

Olfactory integration is important for survival in a natural habitat. However, how the nervous system processes signals of two odorants present simultaneously to generate a coherent behavioral response is poorly understood. Here, we characterize circuit basis for a form of olfactory integration in *Caenorhabditis elegans*. We find that the presence of a repulsive odorant, 2-nonanone, that signals threat strongly blocks the attraction of other odorants, such as isoamyl alcohol (IAA) or benzaldehyde, that signal food. Using a forward genetic screen, we found that genes known to regulate the structure and function of sensory neurons, *osm-5* and *osm-1*, played a critical role in the integration process. Loss of these genes mildly reduces the response to the repellent 2-nonanone and disrupts the integration effect. Restoring the function of OSM-5 in either AWB or ASH, two sensory neurons known to mediate 2-nonanone-evoked avoidance, is sufficient to rescue. Sensory neurons AWB and downstream interneurons AVA, AIB, RIM that play critical roles in olfactory sensorimotor response are able to process signals generated by 2-nonanone or IAA or the mixture of the two odorants and contribute to the integration. Thus, our results identify redundant neural circuits that regulate the robust effect of a repulsive odorant to block responses to attractive odorants and uncover the neuronal and cellular basis for this complex olfactory task.

## Author summary

In their natural environment, animals, including humans, encounter complex olfactory stimuli. Thus, how the brain processes multiple sensory cues to generate a coherent behavioral output is critical for the survival of the animal. In the present study, we combined molecular cellular genetics, optical physiology and behavioral analysis to study a common olfactory phenomenon in which the presence of one odorant blocks the response to another. Our results show that the integrated response is regulated by redundant neuronal circuits that engage several interneurons essential for olfactory sensorimotor responses, a mechanism that likely ensures a robust behavioral response to sensory cues representing information critical for survival.

## Introduction

Odorants represent a wide range of environmental conditions, such as the presence of food, a mate, or a predator. Thus, odorant sensation regulates behaviors that are critical for survival [1]. The mechanisms through which the nervous system detects an olfactory cue, processes the sensory information, and directs the subsequent behaviors have been characterized in different organisms. Most of these studies use volatile chemicals that are individually presented to signal a simplified environmental condition that is either attractive or repulsive [2–6]. However, attractive and repulsive cues often co-exist under a natural condition where an animal has to make a behavioral decision based on these conflicting messages that respectively signal rewards and threats. Thus, characterizing how the nervous system processes complex odorant information is fundamental for our understanding of how the olfactory system operates.

Previous psychophysics studies on human olfaction show that simultaneous presence of two odorants can evoke a response that is different from simple addition of the responses to the individual odorants [7], suggesting complex olfactory processes that integrate the sensory information from two different odorant components. While studies on humans and other mammals pioneered the psychophysical analyses of integrated responses to complex olfactory tasks, studies using simple animals, such as *Drosophila melanogaster* and *Caenorhabditis elegans*, utilize complementary approaches to address underlying molecular and cellular basis [8–13]. The nervous systems of these organisms are highly accessible by genetics, which allows the dissection of signaling pathways and neural circuits that regulate odorant-guided behaviors in complex olfactory environments. For example, a fermenting fruit releases multiple odorants, including attractive odorants that signal food and aversive CO_2_, that are detected by fruit flies. A previous study shows that a mushroom body lobe region in the central nervous system of a fruit fly integrates the food odorant—elicited signals to suppress the CO_2_—evoked output signals. This integration allows the fly to approach food even when the innately repulsive CO_2_ is present [12]. *C*. *elegans* is strongly attracted by an odorant isoamyl alcohol (IAA) that signals food; however, the chemotactic response to IAA, and several other odorants, can be disrupted by the presence of an arthropod repellent DEET (*N*,*N-*diethyl-*meta-*toluamide), which interrupts chemotaxis by activating a G-protein coupled receptor (GPCR) *str-217* in a pair of chemosensory neurons ADL [13]. In addition, the attraction of *C*. *elegans* to the odorant diacetyl can also be blocked by a barrier of a hyperosmotic solution. It is shown that tyramine released by an interneuron potentiates an osmosensory neuron that triggers avoidance in response to hyperosmotic stimulation. Suppressing the pathway through food deprivation motivates worms to cross the hyperosmotic barrier in order to reach the food odorant [11]. Together, these studies demonstrate that the nervous systems of simple organisms use fundamental signaling mechanisms to modulate function of neural circuits to generate a coherent behavioral response when facing sensory cues of different valences.

In this study, we use *C*. *elegans* to characterize molecular and neuronal basis of olfactory integration. With a compact and well-defined nervous system [14], *C*. *elegans* responds to a large number of attractive or repulsive odorants. Several pairs of sensory neurons detect these odorants using GPCRs and downstream cGMP-mediated signaling pathways [2,15–21]. Among well-studied olfactory cues, we chose 2-nonanone and isoamyl alcohol (IAA) to make a pair of odorants with conflicting valences. 2-nonanone is a strong repellent that is sensed by olfactory sensory neurons AWB and ASH. A direct contact with a solution of a high concentration of 2-nonanone kills a worm acutely. Thus, 2-nonanone likely represents a threat to survival [4,22–24]. In contrast, IAA at a wide range of concentrations strongly attracts a worm and is mainly detected by AWC sensory neurons [2,25–27]. In our behavior paradigm, we put 2-nonanone and IAA side-by-side to stimulate worms with an attractive odorant and a repulsive odorant simultaneously. We find that while IAA alone strongly attracts the worms and 2-nonanone alone strongly repels them, IAA present with 2-nonanone at several high concentrations repel the worms as much as 2-nonanone alone. The attractive effect of IAA is completely blocked by 2-nonanone. By screening for mutants that were generated by random mutagenesis, we identified three genes, *osm-5*, *osm-1*, and *dyf-7*, which led us to uncover the circuit mechanisms underlying the integrated response to the repulsive and the attractive odorants.

## Results

### A new behavior paradigm to characterize olfactory integration

**Fig 1:**
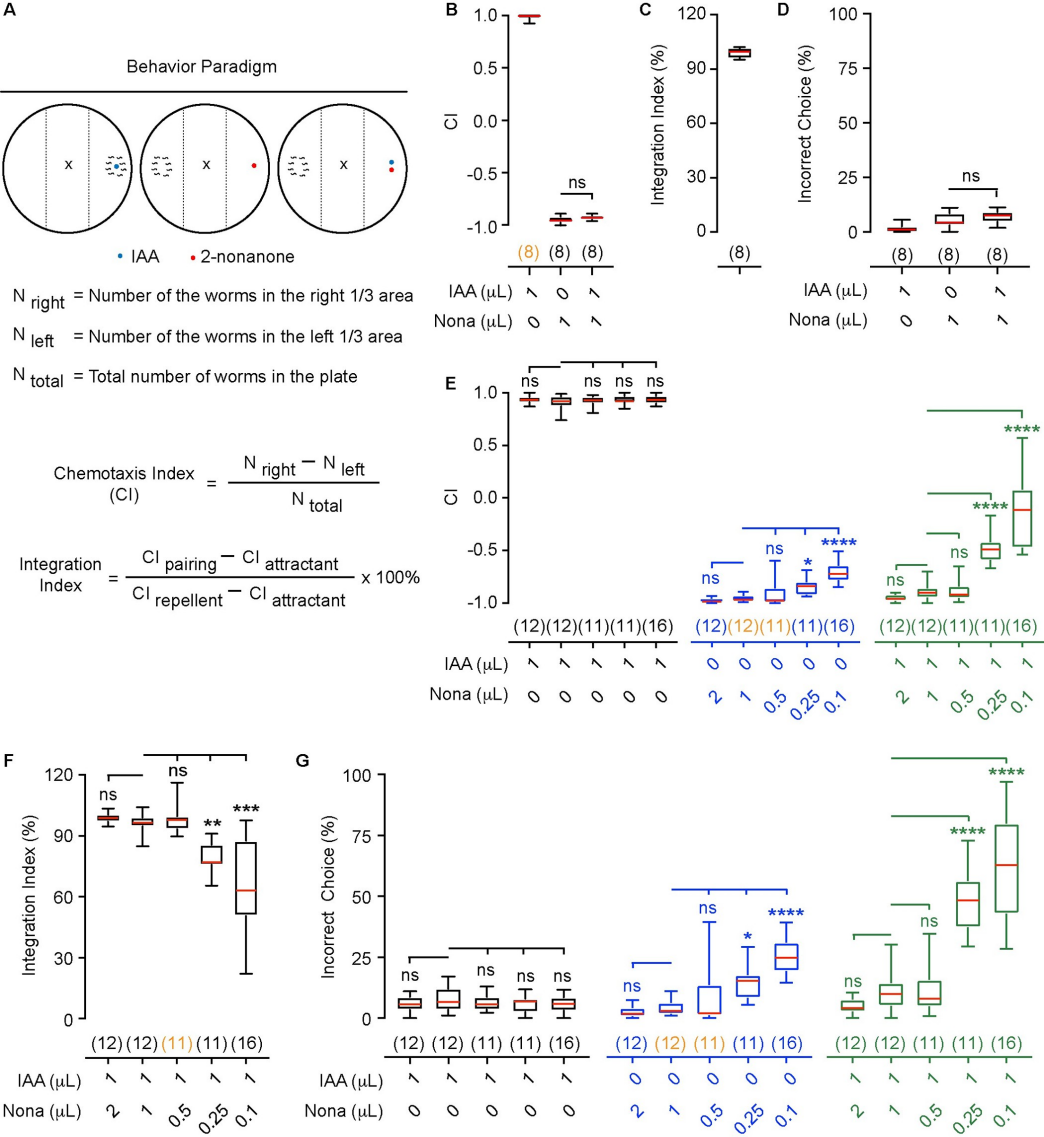
*C*. *elegans* displays olfactory integration. **(A)** A schematic showing chemotaxis assays and definition of choice index and integration index. **(B, C)** The odorant isoamyl alcohol (IAA) attracts wild-type *C*. *elegans*, while the odorant 2-nonanone (Nona) repels it (**B**). However, pairing 2-nonanone with IAA repels *C*. *elegans* similarly as 2-nonanone (**B**), completely blocking the attraction of IAA (**C**). **(D)** The percentages of incorrect choices (defined in Materials and Methods) for assays in **B** and **C. (E—G)** 2-nonanone blocks the attraction of IAA in a dosage-dependent manner. In **B–G**, box plots indicate median, the first and the third quartile, and the minimal and maximal values; the numbers of assays are indicated in the parentheses, which are highlighted in orange if the data are not normally distributed; Nona, 2-nonanone. Two tailed unpaired *t*-test (**B, D,** data are normally distributed) or One way ANOVA with Dunnett’s multiple comparisons test (**E–G,** if data are normally distributed) or Kruskal-Wallis test with Dunn’s multiple comparisons test (**E–G,** if data are not normally distributed), **** *p* < 0.0001, *** *p* < 0.001, ** *p* < 0.01, * *p* < 0.05, ns, not significant.

To study mechanisms underlying olfactory integration, we developed a behavior paradigm based on the standard chemotaxis assay (Fig 1A). We found that consistent with previous findings IAA was strongly attractive and 2-nonanone was strongly repulsive. Strikingly, when these two odorants were placed aside each other, they together repelled wild-type animals as strongly as 2-nonanone alone (Fig 1B and 1C). To quantify the effect of 2-nonanone on the attractive response to IAA, we first separately measured the choice index (CI) for 1 μL IAA (CI_Attractant_) or 1 μL 2-nonanone (CI_Repellent_) and the choice index when worms were presented with 1 μL IAA and 1 μL 2-nonanone together (CI_Pairing_) as shown in Fig 1A. A positive choice index indicates attraction towards the tested odorant and a negative choice index indicates avoidance. We then calculated the integration index, which was defined as the ratio in percentage of the difference between CI_Pairing_ and CI_Attractant_ to the difference between CI_Repellent_ and CI_Attractant_. Thus, the integration index in this study indicates how much the presence of a repellent, such as 2-nonanone, blocks the attraction of an attractant, such as IAA (Fig 1A and Materials and Methods). Using this paradigm, we found that IAA generated a CI close to 1. In comparison, 2-nonanone generated a CI close to -1 (Fig 1B). Strikingly, pairing the two odorants generated a CI close to -1 (Fig 1B and 1C). Thus, we observed an integration index about 100%, indicating a near complete blocking of IAA attraction by 2-nonanone. Because presence of 1 μL 2-nonanone and 1 μL IAA evoked a response different from a simple addition of the response to 1 μL 2-nonanone and the response to 1 μL IAA, which would have yielded a CI of close to 0, we used this behavioral paradigm to characterize olfactory integration.

To better understand how the indexes were produced by behavioral choices made by the animals in these assays, we also quantified the percentage of incorrect choice made by the worms in each chemotaxis assay (Materials and Methods). We found that less than 8% of wild-type animals made incorrect choices in all three behavior assays (Fig 1D). Thus, the presence of 1 μL 2-nonanone together with 1 μL IAA is strongly repulsive. To further characterize the effect of 2-nonanone on IAA attraction, we paired 1μL IAA with a series of different amounts of 2-nonanone ranging from 0.1 μL to 2 μL (Fig 1E–1G). We found that 2, 1 or 0.5 μL 2-nonanone significantly blocked the attraction of 1μL IAA, demonstrated by repulsion of the worms by the paired odorants, and that 0.25 or 0.1 μL 2-nonanone showed much reduced repulsion when paired with 1 μL IAA (Fig 1E–1G). These results indicate that the repellent 2-nonanone blocks the attraction of IAA in a dosage-dependent manner. We characterize the mechanism of integration using 1 μL 2-nonanone paired with 1 μL IAA unless otherwise indicated.

### A forward genetic screen identified 3 mutants that are defective in olfactory integration

**Fig 2:**
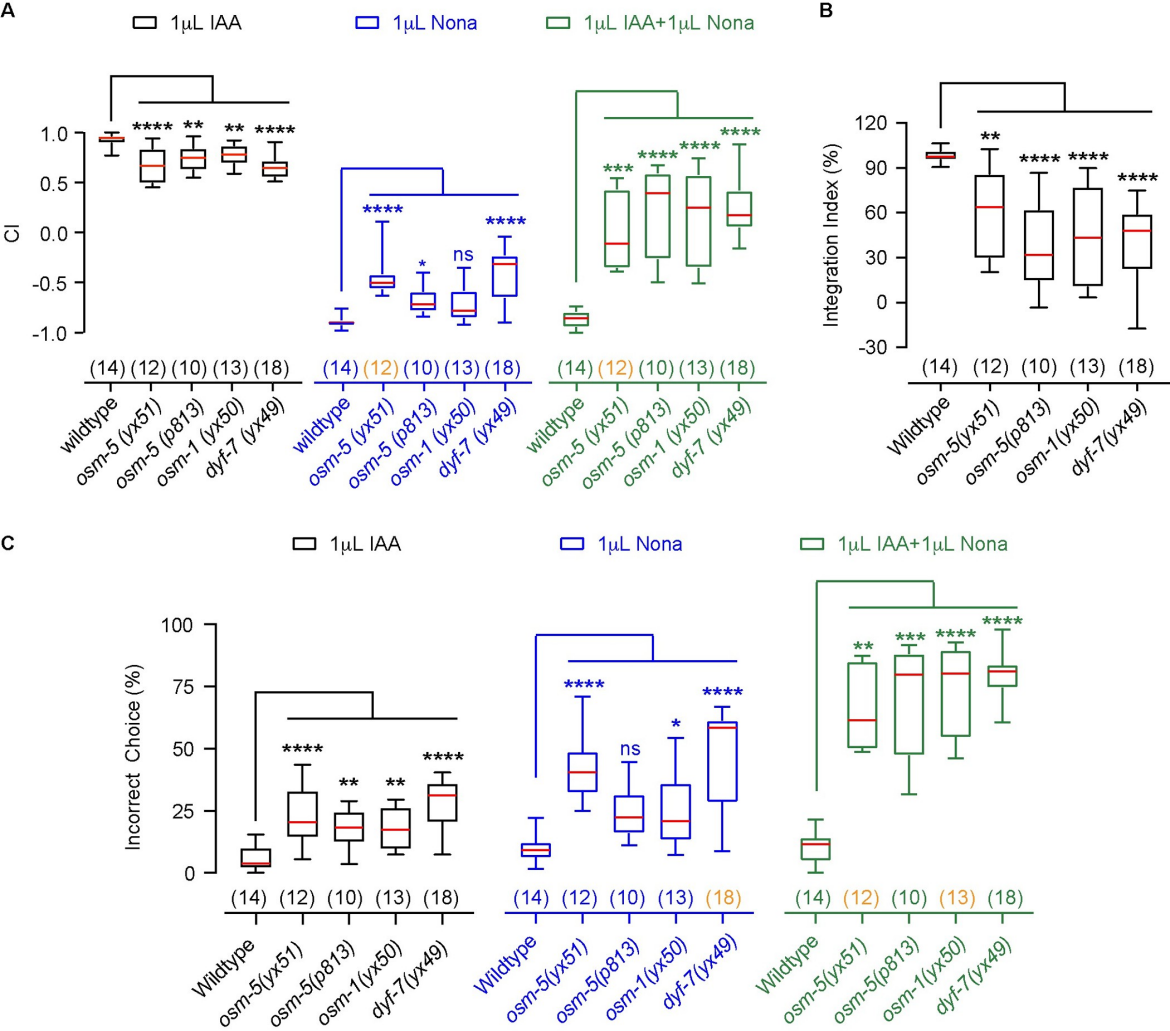
Mutations in genes regulating sensory neurons disrupt olfactory integration. **(A, B)** Mutations in *osm-5*, *osm-1* and *dyf-7* mildly disrupt chemotaxis to IAA or 2-nonanone (**A**), but strongly disrupt olfactory integration (**A, B**). **(C)** The percentages of incorrect choices for assays in **A, B**. In **A–C**, box plots indicate median, the first and the third quartile, and the minimal and maximal values; the numbers of assays are indicated in the parentheses, which are highlighted in orange if the data are not normally distributed; Nona, 2-nonanone. Mutant indexes or percentages were compared with wild type using One way ANOVA with Dunnett’s multiple comparisons test (if data are normally distributed) or Kruskal-Wallis test with Dunn’s multiple comparisons test (if data are not normally distributed), **** *p* < 0.0001, *** *p* < 0.001, ** *p* < 0.01, * *p* < 0.05, ns, not significant.

To understand the regulation of olfactory integration, we conducted a forward genetic screen. We generated a library of mutants by using EMS-based random mutagenesis and screened for F2 clones that were capable of sensing both IAA and 2-nonanone separately, but significantly defective for the integrated response to the presence of 1 μL 2-nonanone paired with 1 μL IAA (Materials and Methods). Using these criteria, we identified 3 mutants, *yx51*, *yx50* and *yx49* (Fig 2), among ~ 20,000 F2 clones generated by EMS-mutagenesis.

All three mutant strains, *yx51*, *yx50*, and *yx49*, showed slightly reduced chemotaxis to IAA (Fig 2A), weaker avoidance of 2-nonanone (Fig 2A), and significantly reduced avoidance of the mixture and integration indexes (Fig 2A and 2B). Because integration index measures how much pairing with 2-nonanone changes chemotaxis to IAA relative to the difference between the chemotaxis to each of the odorants (Fig 1A), these results indicate that *yx51*, *yx50* and *yx49* are defective in olfactory chemotaxis and olfactory integration. These three mutant strains also showed dramatically increased portions of animals that made incorrect choices when tested in chemotaxis and olfactory integration (Fig 2C), indicating that these mutant animals displayed attraction towards IAA despite the presence of 2-nonanone, which results in defects in generating the integrated response to 2-nonanone paired with IAA (Fig 2C).

### *osm-5* and *osm-1* regulate olfactory integration

**Fig 3:**
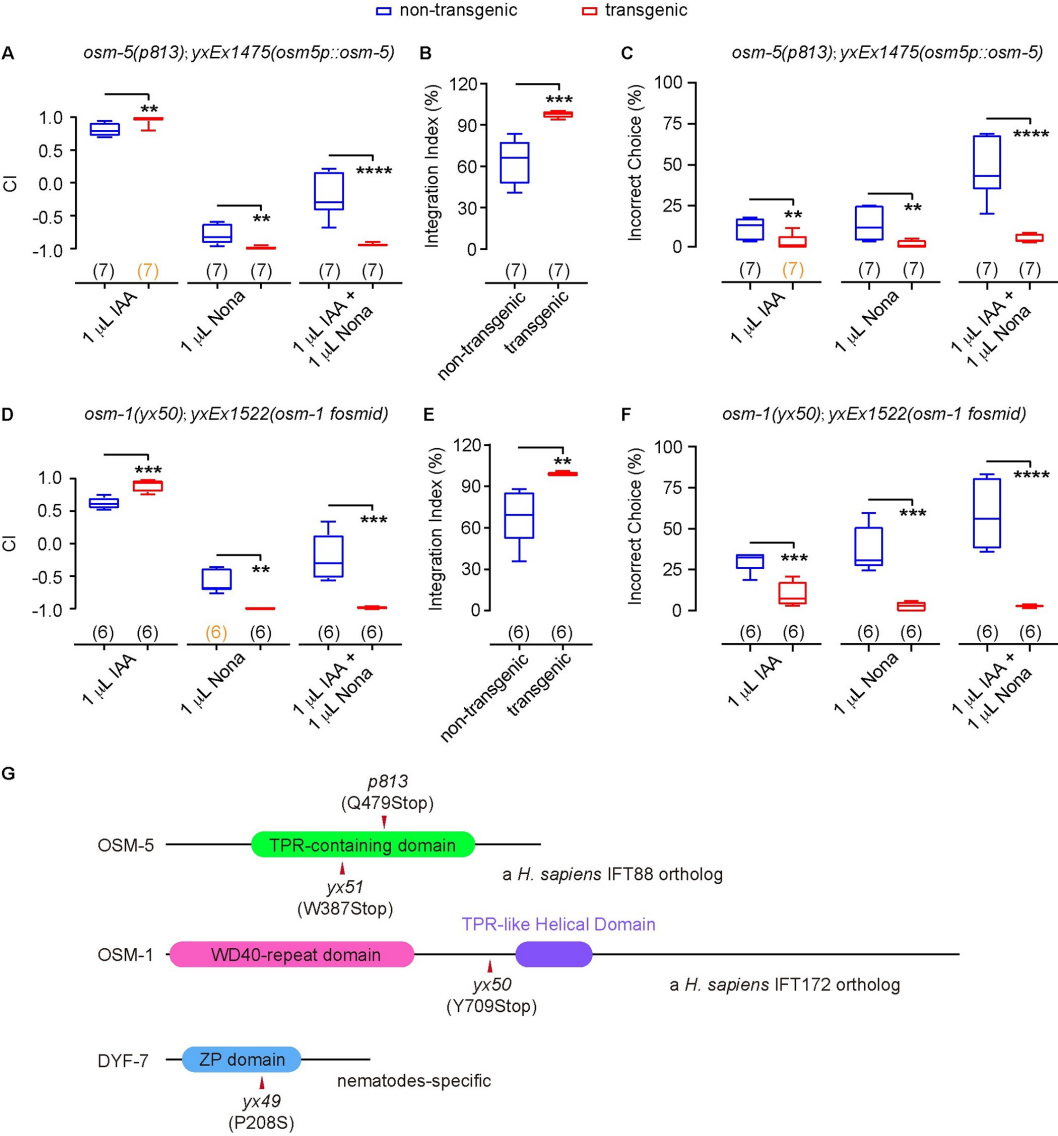
Expressing *osm-5 or osm-1* rescues the defects in olfactory integration. **(A—C)** Expressing a wild-type *osm-5* cDNA using an *osm-5* promoter rescues the defects of *osm-5(p813)* mutants in chemotaxis to IAA or 2-nonanone (**A**) and olfactory integration (**A, B**), as well as behavioral choices during the assays (**C**). **(D—F)** Expressing a wild-type fosmid containing *osm-1* genomic sequence rescues the defects of *osm-1(yx50)* mutants in chemotaxis to IAA or 2-nonanone (**D**) and olfactory integration (**D, E**), as well as behavioral choices during the assays (**F**). **(G)** Schematics showing protein domains of OSM-5, OSM-1 and DYF-7, as well as the mutations identified in this or previous studies. In **A–F,** box plots indicate median, the first and the third quartile, and the minimal and maximal values; the numbers of assays are indicated in the parentheses, which are highlighted in orange if the data are not normally distributed; Nona, 2-nonanone. Two tailed unpaired *t*-test (if data are normally distributed) or two tailed Mann-Whitney test (if data are not normally distributed) is used to compare transgenic animals and their non-transgenic siblings tested in parallel. **** *p* < 0.0001, *** *p* < 0.001, ** *p* < 0.01, * *p* < 0.05.

To identify genetic lesions that disrupted olfactory integration in *yx51*, *yx50* and *yx49* mutant animals, we sequenced the genomes of all three mutant strains, which identified a list of candidate mutations in each strain. We confirmed the causal mutations by testing the rescuing effect of expressing wild-type candidate genes on olfactory chemotaxis and olfactory integration (Figs 3A–3F and S1). We found that the phenotype of *yx51* was rescued by expressing wild-type *osm-5* cDNA sequence driven by an *osm-5* promoter (S1 Fig). Another previously identified mutation in *osm-5*, *p813*, showed phenotypes similar to that of *yx51*. *p813* was also rescued by the same DNA construct of *osm-5* (Fig 3A–3C). These results together indicate that the G to A mutation that changes tryptophan 387 of OSM-5 to a stop codon (Fig 3G) disrupted olfactory integration in *yx51* mutant animals. Expressing fosmid WRM0638dE09 that contains the genomic region of *osm-1* rescued the mutant phenotypes of *yx50* (Fig 3D–3F). WRM0638dE09 also contains another two genes, *pat-9* and *k08b5*.*2*. However, our whole-genome sequencing analysis did not identify any mutation in the coding region of either of these two genes. Based on all these results, we concluded that the C to A mutation that changed tyrosine 709 of OSM-1 to a stop codon (Fig 3G) generated the defect in olfactory integration in *yx50* mutant animals.

Previous studies show that both *osm-5* and *osm-1* encode intraflagellar transport proteins that regulate the generation and function of sensory cilia [28–30]. We speculated that the candidate gene in *yx49* mutant animals may also be related to sensory neurons. Thus, we performed dye-filling assay, which assessed the function of sensory cilia. We found that *yx49*, *yx50* and *yx51* were all defective in dye-filling (S2 Fig), revealing the impaired cilia in these mutant animals. Our sequencing analysis on *yx49* identified a C to T mutation that changed proline 208 of DYF-7 to serine (Fig 3G). *dyf-7* encodes an extracellular matrix protein that regulates the anchoring of dendrite tip during the development of sensory neurons [31]. These results suggest that disfunction of DYF-7 in *yx49* may account for the reduced olfactory integration behavior in this mutant strain. Together, our study identified genes that are known to regulate cilia structure and function or development of sensory neurons. Similar to previously characterized mutations in these genes [2,32,33], *osm-5(yx51)*, *osm-1(yx50)*, and *dyf-7(yx49)* are defective in olfactory chemotaxis (Fig 2A). Our studies show that these mutant animals are also defective in olfactory integration.

### AWB and ASH neurons function redundantly to regulate olfactory integration

**Fig 4:**
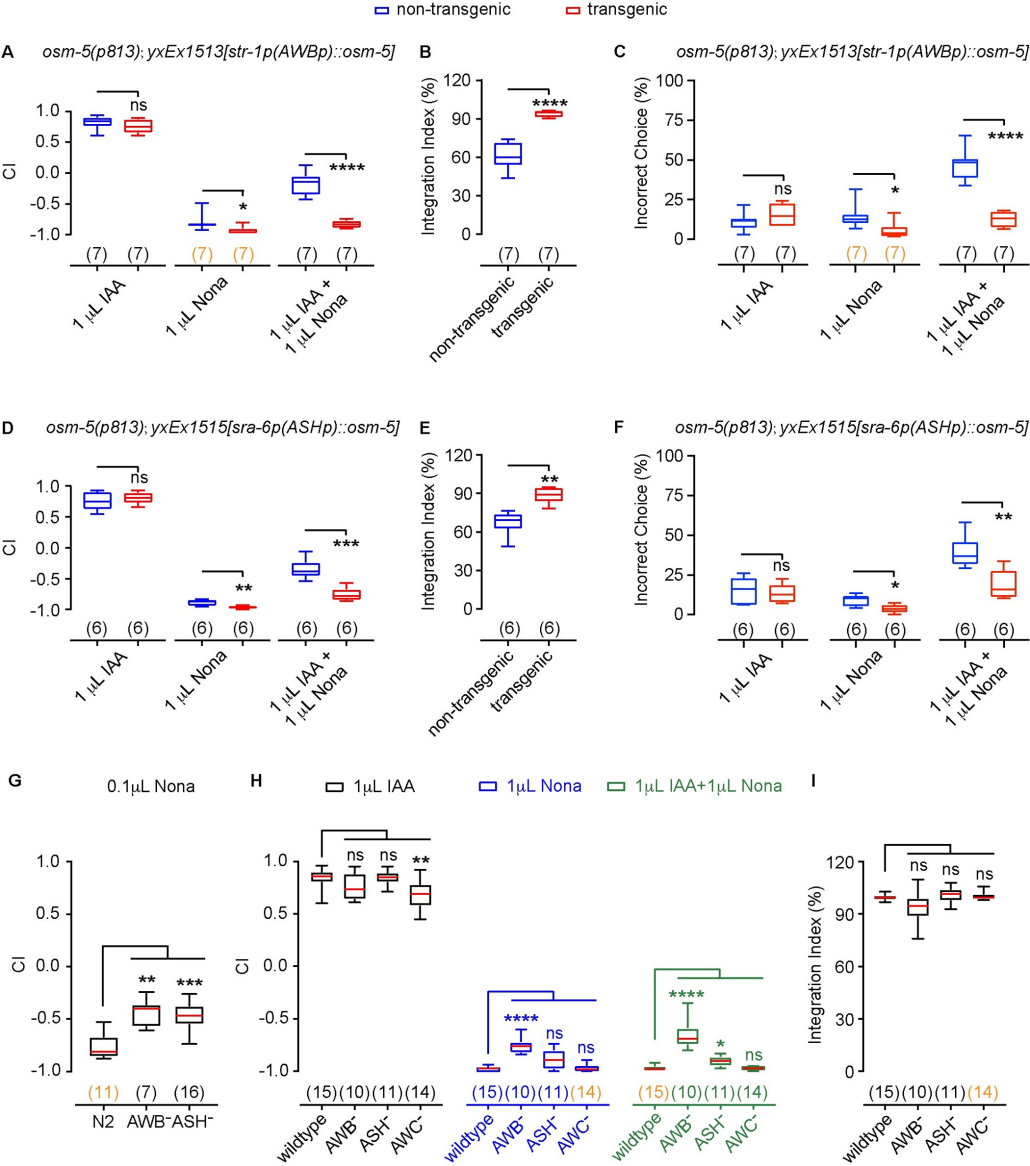
*osm-5* acts in AWB or ASH neurons to regulate chemotaxis and olfactory integration. **(A—C)** Expressing a wild-type *osm-5* cDNA in AWB rescues the defects of *osm-5(p813)* mutants in chemotaxis to 2-nonanone (Nona) (**A**) and olfactory integration (**A, B**), as well as behavioral choices during the assays (**C**). **(D—F)** Expressing a wild-type *osm-5* cDNA in ASH rescues the defects of *osm-5(p813)* mutants in chemotaxis to 2-nonanone (Nona) (**D**) and olfactory integration (**D, E**), as well as behavioral choices during the assays (**F**). **(G)** Ablation of AWB or ASH impairs avoidance of 0.1 μL 2-nonanone. **(H, I)** Ablation of AWC reduces attraction of 1 μL IAA, ablation of AWB reduces avoidance of 1 μL 2-nonanone, ablation of AWB or ASH reduces avoidance of the mixture of 1 μL 2-nonanone and 1 μL IAA (**H**) without a significant effect on the integration index (**I**). In **A–I,** box plots indicate the median, the first and the third quartile, and the minimal and maximal values; the numbers of assays are indicated in the parentheses, which are highlighted in orange if the data are not normally distributed. In **A–F,** two tailed unpaired *t*-test (if data are normally distributed) or two tailed Mann-Whitney test (if data are not normally distributed) is used to compare transgenic animals and their non-transgenic siblings tested in parallel. In **G–I,** indexes of ablated animals are compared with that of wild type (N2) using One way ANOVA with Dunnett’s multiple comparisons test (if data are normally distributed) or Kruskal-Wallis test with Dunn’s multiple comparisons test (if data are not normally distributed). **** *p* < 0.0001, *** *p* < 0.001, ** *p* < 0.01, * *p* < 0.05, ns, not significant.

To characterize the mechanism of olfactory integration, we sought the underlying neural circuit. Because two independently generated *osm-5* mutant alleles showed similar phenotypes, we focused on the analysis of *osm-5* hereafter. *osm-5* is expressed in ciliated sensory neurons [28]. Because *osm-5(p813)* animals were defective in DiO staining (S2 Fig, Materials and Methods), which stains several neurons that are exposed to the environment with a green fluorescent dye, *osm-5* may function in the DiO labeled amphid chemosensory neurons (ADL, ASH, ASI, ASJ, ASK, AWB) to regulate olfactory integration. We tested AWB and ASH neurons, because they were shown to respond to 2-nonanone and to mediate 2-nonanone-induced repulsion [4,22,23]. We expressed a wild-type *osm-5* cDNA selectively in AWB or ASH in the *osm-5(p813)* mutant animals using cell-specific promoters (Materials and Methods). We found that expressing wild-type *osm-5* in either AWB neurons using the *str-1* promoter [4] or in ASH neurons using the *sra-6* promoter [34] did not change the chemotaxis to IAA, but rescued the chemotaxis to 2-nonanone, resulting in the rescue of olfactory integration (Figs 4A–4F and S3). Although *sra-6* is also expressed in several other sensory neurons in addition to ASH [35], these neurons are not known to mediate 2-nonanone response. These results suggest that strengthening the avoidance of 2-nonanone through the function of AWB or ASH increases the blocking effect on IAA.

To further assess the role of AWB and ASH neurons in blocking IAA attraction, we tested the effects of ablating these neurons. We found that ablating either AWB or ASH reduced the avoidance of 0.1 μL 2-nonanone (Fig 4G), consistent with the critical role of AWB and ASH in chemotaxis of 2-nonanone. Ablating AWB reduced chemotaxis to 1 μL 2-nonanone, but ablating ASH had no effect on chemotaxis to 1 μL 2-nonanone (Fig 4H). Meanwhile, ablating AWC reduced chemotaxis to 1μL IAA (Fig 4H), also consistent with the role of AWC in regulating IAA-evoked attraction [2]. The small defects in chemotaxis generated by ablations are likely due to the high concentrations of the odorants used in these experiments and potential redundant roles of AWB and ASH in mediating 2-nonanone evoked avoidance. Nevertheless, no significant defect in integrated response was observed in worms with ablated AWB or ASH or AWC neurons (Fig 4I). Together these results supported the redundant role of AWB and ASH in regulating olfactory integration.

### The AWB neurons integrate sensory responses to IAA and 2-nonanone

**Fig 5:**
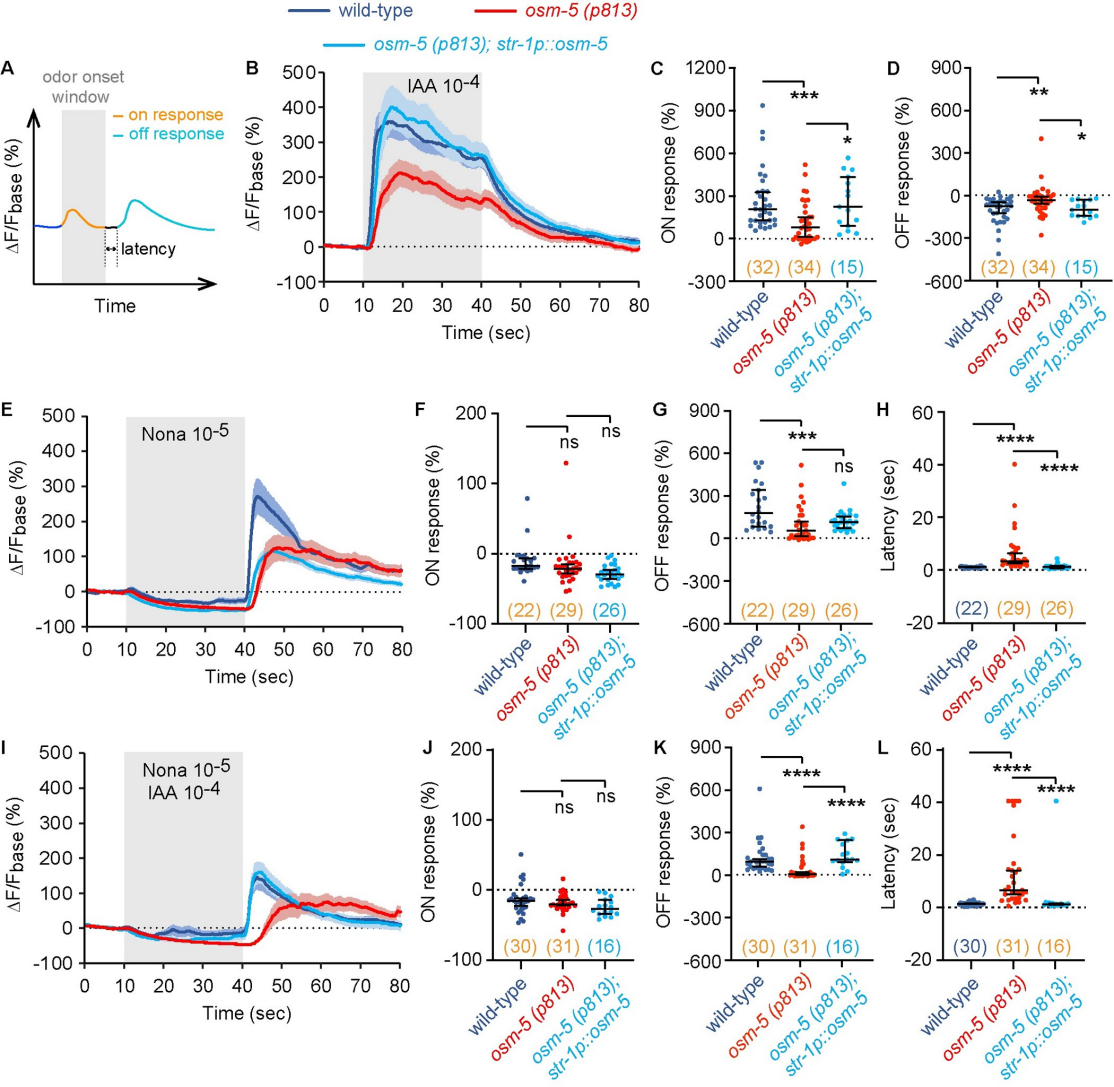
*osm-5* acts in AWB to regulate neuronal responses of AWB to IAA, 2-nonanone, and the mixture. **(A)** Schematics illustrating the definition of ON and OFF response and the latency of the OFF response. **(B—D)** Exposure to IAA increases GCaMP signal of AWB (ON response) and removal of IAA decreases it (OFF response). The *osm-5(p813)* mutation disrupts both the ON and OFF responses and expressing *osm-5* in AWB rescues the defects. **(E—H)** Exposure to 2-nonanone (Nona) suppresses the GCaMP signal of AWB (ON response) and removal of 2-nonanone increases it (OFF response). The *osm-5(p813)* mutation decreases the amplitude and increases the latency of the OFF response and expressing *osm-5* in AWB rescues the defect in latency. **(I—L)** Exposure to the mixture of IAA and 2-nonanone (Nona) suppresses the GCaMP signal of AWB (ON response) and removal of the mixture increases it (OFF response). The *osm-5(p813)* mutation decreases the amplitude and increases the latency of the OFF response and expressing *osm-5* in AWB rescues both defects. The change in fluorescence intensity (ΔF) for each frame is the difference between its fluorescence intensity and the average intensity over the 10-second recording before the stimulus onset (F_base_): ΔF = F—F_base_. The average ΔF/F_base_ % during the 10-second window after onset minus average ΔF/F_base_ % of the 10-second window before onset is used to measure ON response_._ The average ΔF/F_base_ % during the 10-second window after removal minus average ΔF/F_base_ % of the 10-second window before removal is used to measure OFF response. Latency is defined as the time that it takes for the calcium signal to rise to the mean of the 10-second window before odor removal plus 3 × standard deviation. In **B, E, I**, solid lines and shades are respectively mean and SEM and in **C, D, F–H, J–L**, horizontal bars in each graph are median with 95% confidence interval, individual data points are shown as dots. The numbers of the worms imaged are shown in the parentheses, which are highlighted in orange if the data are not normally distributed. Kruskal-Wallis Test with Dunn’s multiple comparisons test is used to compare mutants with wild-type and rescued animals, because data are not normally distributed. **** *p* < 0.0001, *** *p* < 0.001, ** *p* < 0.01, * *p* < 0.05, ns, not significant.

Next, to understand how AWB neurons regulate olfactory integration, we measured the response of AWB to IAA or 2-nonanone alone and to the mixture of the two odorants using transgenic animals that selectively expressed a calcium sensor GCaMP6s [36] in AWB. We used the amplitude of the ON and OFF responses and the latency of the OFF response as the indexes to evaluate calcium signals (Fig 5A). First, we found that exposure to IAA activated AWB, indicated by a rapidly increased GCaMP6 signal that lasted during the presence of IAA stimulation. Removal of IAA led to a gradual decrease in the GCaMP6 signal in AWB (Fig 5B–5D). Consistent with previous findings, exposure to 2-nonanone modestly suppressed AWB activities and removal of 2-nonanone activated AWB (Fig 5E–5H). Strikingly, the mixture of IAA and 2-nonanone generated AWB GCaMP6 signal with a pattern comparable to that generated by 2-nonanone alone (Fig 5I–5L), showing that at the level of AWB neuronal activities, presence of 2-nonanone blocks the effect of IAA stimulation.

Next, we examined the difference between wild type and *osm-5* mutants. First, we found that the amplitude of AWB response to IAA was much lower in *osm-5* than in wild-type, which was rescued by expressing *osm-5* in AWB (Fig 5B–5D). However, the chemotactic response to IAA was not affected by expressing *osm-5* in AWB (Fig 4A). Therefore, the response of AWB to IAA is unlikely to play a critical role in regulating attraction to IAA, consistent with the critical role of AWC sensory neurons in mediating the attraction to IAA [25,27]. Second, we found that the OFF response of AWB to 2-nonanone, *i*.*e*. the response to the removal of 2-nonanone, displayed a reduced amplitude and an increased latency in *osm-5* mutant animals (Fig 5E–5H). Expressing *osm-5* in AWB significantly rescued the defect in the latency, but not the defect in amplitude (Fig 5E–5H). Expressing *osm-5* in AWB was sufficient to rescue chemotactic avoidance of 2-nonanone (Figs 4A–4C and S3A–S3C). Thus, the mutation in *osm-5* alters 2-nonanone avoidance by increasing the latency of 2-nonanone-evoked OFF response in AWB. This finding is consistent with a previous study showing that AWB integrates its response to the changes in odorant concentration over time [23]. Third, we found that when stimulated with a mixture of IAA and 2-nonanone, AWB in *osm-5(p813)* also displayed a decreased amplitude and an increased latency in response to the removal of the stimuli, both of which, as well as the olfactory integrated response in behavior, were rescued by *osm-5* expression in AWB (Figs 5I–5L, 4A–4C and S3A–S3C). These results together demonstrate that the latency and the amplitude of AWB OFF response regulate olfactory integration.

### ASH neurons respond to 2-nonanone, but not to IAA

**Fig 6:**
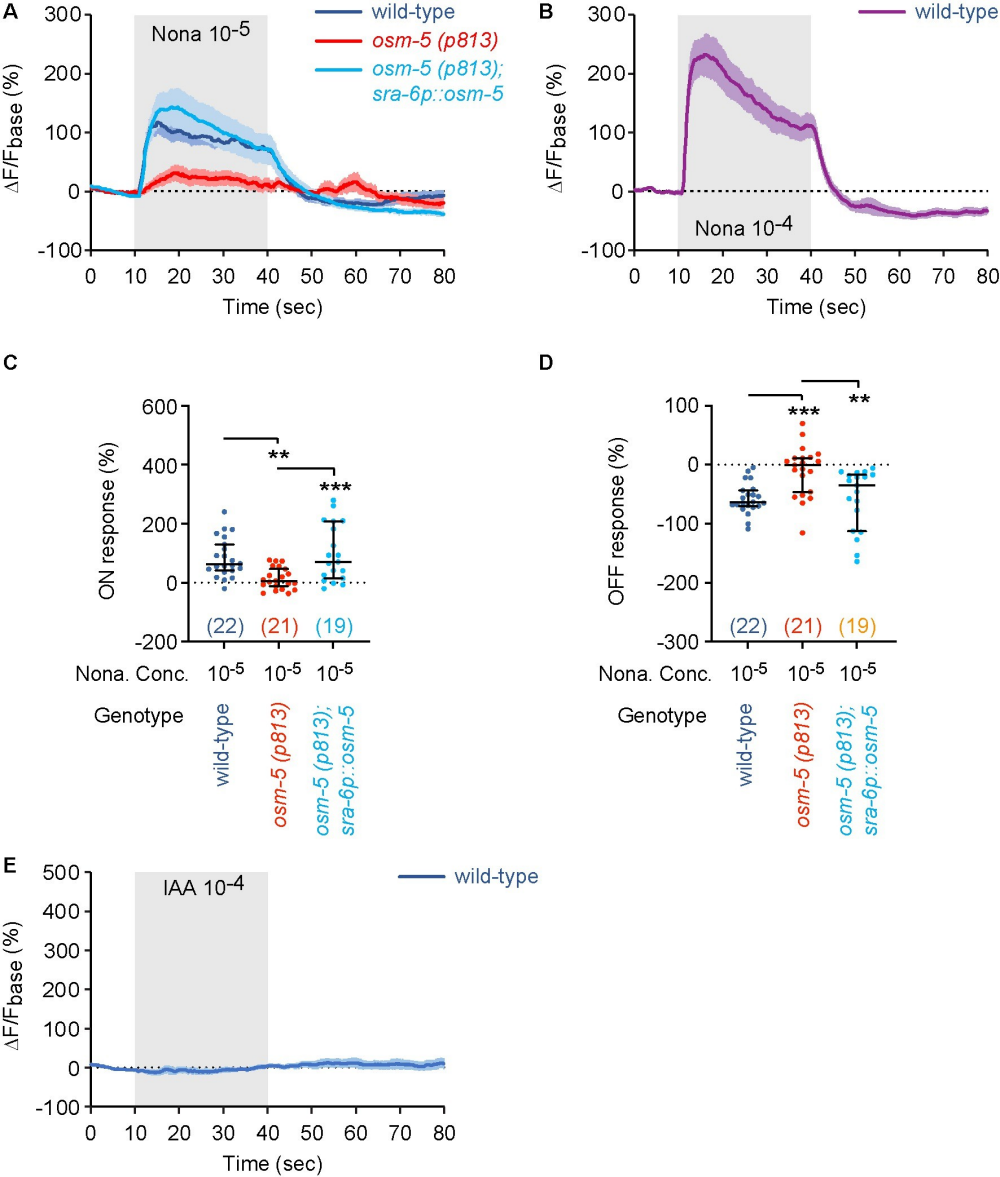
*osm-5* acts in ASH to regulate neuronal response of ASH to 2-nonanone. **(A—D)** Exposure to 2-nonanone (Nona) increases GCaMP signal of ASH (ON response) and removal of 2-nonanone decreases it (OFF response). The *osm-5(p813)* mutation disrupts both the ON and OFF responses and expressing *osm-5* in ASH rescues the defects. **(E)** Exposure to IAA does not induce a significant change in GCaMP signal of ASH. The change in fluorescence intensity (ΔF) for each frame is the difference between its fluorescence intensity and the average intensity over the 10-second recording before the stimulus onset (F_base_): ΔF = F—F_base_. The average ΔF/F_base_ % during the 10-second window after onset minus average ΔF/F_base_ % of the 10-second window before onset is used to measure ON response_._ The average ΔF/F_base_ % during the 10-second window after removal minus average ΔF/F_base_ % of the 10-second window before removal is used to measure OFF response. In **A, B, E,** solid lines and shades are respectively mean and SEM and in **C, D,** horizontal bars in each graph are medianwith 95% confidence interval, individual data points are shown as dots. The numbers of the worms imaged are shown in the parentheses, which are colored in orange if the data are not normally distributed. Difference among the groups is analyzed by One way ANOVA with Dunnett’s multiple comparisons test (if data are normally distributed) or Kruskal-Wallis test with Dunn’s multiple comparisons test (if data are not normally distributed). *** *p* < 0.001, ** *p* < 0.01, ns, not significant.

Previous work using calcium imaging showed that ASH acutely responded to the increase or decrease in the concentration of 2-nonanone [23]. Similarly, we found that exposure to 2-nonanone evoked a rapid increase in the GCaMP6 signal of ASH and removal of 2-nonanone triggered a rapid decrease (Fig 6A–6D). In contrast, exposure to IAA did not generate any detectable GCaMP6 signal in ASH, indicating that ASH are not IAA-responding neurons (Fig 6E). The mutation in *osm-5(p813)* disrupted the responses of ASH to the onset and removal of 2-nonanone. Both of the defects in ASH neuronal responses, as well as the behavioral defects of *osm-5(p813)* mutants in their chemotaxis to 2-nonanone and olfactory integration, were rescued by expressing wild-type *osm-5* in ASH (Figs 6A, 6C, 6D, 4D–4F and S3D–S3F). Together, these results indicate that ASH neurons regulate olfactory integration by responding to 2-nonanone.

### The interneurons AVA, AIB and RIM respond to IAA and 2-nonanone differently

**Fig 7:**
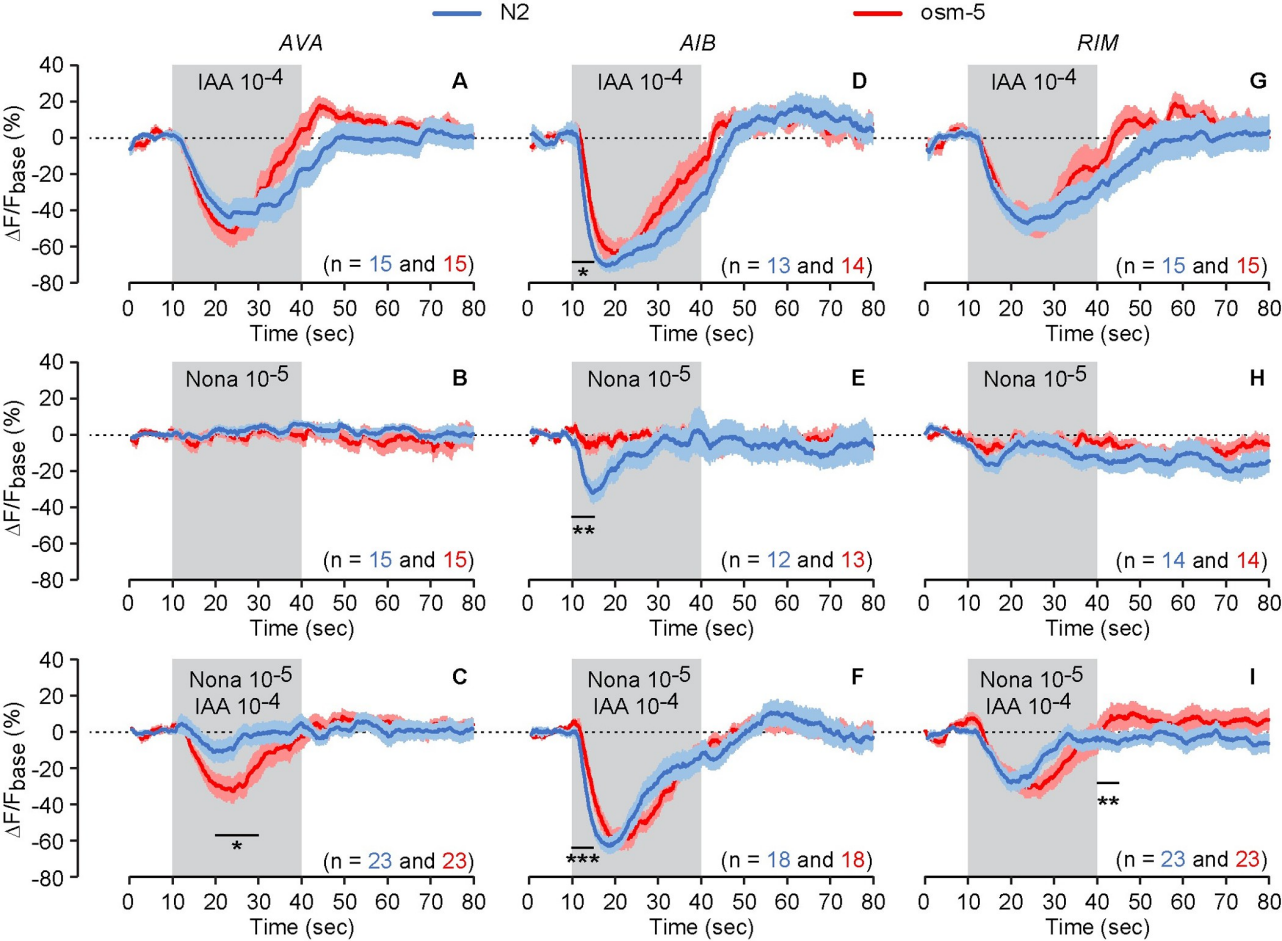
*osm-5* regulates neuronal responses of AVA, AIB and RIM interneurons to IAA, 2-nonanone, and the mixture. **(A—C)** Exposure to 10^−4^ IAA suppresses GCaMP signal of AVA in both wild-type and *osm-5(p813)* mutant animals (**A**). Exposure to 10^−5^ 2-nonanone (Nona) does not evoke a significant GCaMP signal in AVA (**B**). Exposure to 10^−5^ 2-nonanone (Nona) and 10^−4^ IAA suppresses GCaMP signal of AVA in *osm-5(p813)* mutants, but not in wild-type animals (**C**). **(D—F)** Exposure to 10^−4^ IAA suppresses GCaMP signal of AIB in both wild-type and *osm-5(p813)* mutant animals (**D**). Exposure to 10^−5^ 2-nonanone (Nona) suppresses GCaMP signal of AIB in wild-type animals, but not in *osm-5(p813)* mutant animals (**E**). Exposure to 10^−5^ 2-nonanone (Nona) and 10^−4^ IAA suppresses GCaMP signal of AIB in wild-type, and the suppression is reduced in *osm-5(p813)* mutant animals (**F**). **(G—I)** Exposure to 10^−4^ IAA suppresses GCaMP signal of RIM in both wild-type and *osm-5(p813)* mutant animals (**G**). Exposure to 10^−5^ 2-nonanone (Nona) does not evoke a significant GCaMP signal in RIM in either wild-type or *osm-5(p813)* mutant animals (**H**). Exposure to 10^−5^ 2-nonanone and 10^−4^ IAA suppresses the GCaMP signal of RIM in both wild-type and *osm-5(p813)* mutant animals (**I**). The change in fluorescence intensity (ΔF) for each frame is the difference between its fluorescence intensity and the average intensity over the 10-second recording before the stimulus onset (F_base_): ΔF = F—F_base_. The average ΔF/F_base_ % during the 5-second or 2^nd^ 10-second window after onset minus average ΔF/F_base_ % of the 10-second window before onset is used to measure ON response (Materials and Methods)_._ The average ΔF/F_base_ % during the 5-second window after removal minus average ΔF/F_base_ % of the 10-second window before removal is used to measure OFF response. The solid lines and shades are respectively mean and SEM. The numbers of animals imaged are shown on each panel. Detailed statistics is presented in S4 Fig. *** *p* < 0.001, ** *p* < 0.01, * *p* < 0.05.

Our results suggest that 2-nonanone-evoked signals mediated by either AWB or ASH neurons block IAA-evoked signals mediated by AWC neurons to generate olfactory integration observed in this study. We hypothesized that IAA-evoked and 2-nonanone-evoked signals were integrated in neurons immediately downstream to both of AWC and AWB or both of AWC and ASH neurons. To test this hypothesis, we examined sensory-evoked calcium responses in interneurons AIB, AVA and RIM using a transgenic line expressing GCaMP3 in these neurons [37] and stimulated the worms with IAA, 2-nonanone and the mixture of the two odorants. These interneurons receive direct and indirect synaptic inputs from olfactory sensory neurons and their activation generates reversals to regulate chemotactic movements [14,37–39]. First, we found that GCaMP3 signal of AVA neurons was suppressed by the onset of IAA, and did not respond to either 2-nonanone or the odorant mixture in wild-type animals, suggesting that 2-nonanone-evoked signal suppresses IAA-evoked response in AVA (Figs 7A–7C and S4). The mutation of *osm-5* weakens the suppressing effects of 2-nonanone on IAA in AVA. These results together suggest that AVA neurons integrate the neural signals evoked by IAA and 2-nononane and *osm-5* regulates the integration. Second, we found that the GCaMP3 signals of AIB neurons were suppressed by exposure to IAA, 2-nonanone, and the mixture of these two odorants in wild-type animals (Figs 7D–7F and S4). The *osm-5* mutation altered these responses. These observations suggest that AIB neurons process both IAA and 2-nonanone signals and *osm-5* regulates these responses. In addition, we found that the GCaMP3 signals of RIM neurons were suppressed by exposure to IAA or by exposure to the mixture of IAA and 2-nonanone but not by 2-nonanone alone in wild-type animals (Figs 7G–7I and S4). The *osm-5* mutation did not significantly alter the calcium response of RIM. These results together suggest that RIM mainly process IAA evoked signals, which is largely independent of *osm-5*. To further characterize the function of these interneurons, we inhibited the activity of AVA and RIM using a gain-of-function form of a potassium channel *twk-18* [40] or disrupted the function of the neural circuits by expressing a tetanus toxin to block neurotransmission [41] using the *nmr-1* promoter [42]. We also inhibited the function of AIB by expressing *twk-18(gf)* in AIB. We found that expressing *nmr-1p*::*twk-18(gf)* or *nmr-1p*::*TeTx* or *AIBp*::*twk-18(gf)* did not significantly disrupt the integrated response to 2-nonanone and IAA, indicated by the comparable integration indexes in transgenic animals and in their non-transgenic siblings (S5 Fig). These results suggest that although the interneurons AIB, AVA and RIM process sensory signals generated by 2-nonanone and IAA, their activities are not required for olfactory integration, showing redundancy of the underlying neural circuits at the level of interneurons.

### The *osm-5(p813)* mutant animals are defective in several complex olfactory tasks

Finally, we tested how general the *osm-5(p813)* mutant animals were defective in olfactory integration by examining integrated responses to pairing 2-nonanone with another commonly used attractant, benzaldehyde [2]. 0.02 μL benzaldehyde is highly attractive to *C*. *elegans* [43]. We paired 0.02 μL benzaldehyde with 1 μL 2-nonanone to examine chemotactic response in worms and found that 2-nonanone blocked the attraction of 0.02 μL benzaldehyde and generated an integration index more than 90% (S6A and S6B Fig). While *osm-5(p813)* mutant animals showed normal chemotactic behavior towards benzaldehyde and slightly reduced chemotaxis towards 2-nonanone, they are dramatically defective in response to the paired odorants (S6A and S6B Fig). These results indicate that *osm-5(p813)* mutant animals are also defective in olfactory integration of benzaldehyde with 2-nonanone.

Previously, mutants with defective cilia, such as *che-2* or *osm-5*, were shown to be defective in attractive chemotaxis toward diluted IAA, diacetyl and benzaldehyde, and in avoidance of pure benzaldehyde [2,32,44]. How *osm-5* mutants respond to IAA or diacetyl at higher concentrations remains unknown. Increasing the concentration of IAA, diacetyl, or benzaldehyde switches the odorant from being attractive to being repulsive [45]. At lower concentrations, exposure to IAA suppresses AWC calcium activities and removal of IAA activates AWC [27]. At higher concentrations, exposure to IAA activates nociceptive sensory neurons ASH and removal of IAA activates AWB similarly as the repellent 2-nonanone [4,45]. Because the mutation in *osm-5(p813)* disrupted the neuronal response of AWB and ASH to repulsive odorant 2-nonanone and to the mixture of attractive and repulsive odorants, we hypothesized that *osm-5(p813)* also disrupted the repulsion of IAA at higher concentrations. We found that in comparison with wild type, *osm-5(p813)* mutant animals are more attracted to not only 5μL benzaldehyde, which is consistent with previous study [2], but also to 10μL IAA, or 10μL diacetyl (S6C–S6E Fig). These results indicate that *osm-5(p813)* also disrupts concentration-dependent chemotaxis towards odorants. Together, these results suggest that OSM-5 regulates several complex olfactory tasks.

## Discussion

**Fig 8:**
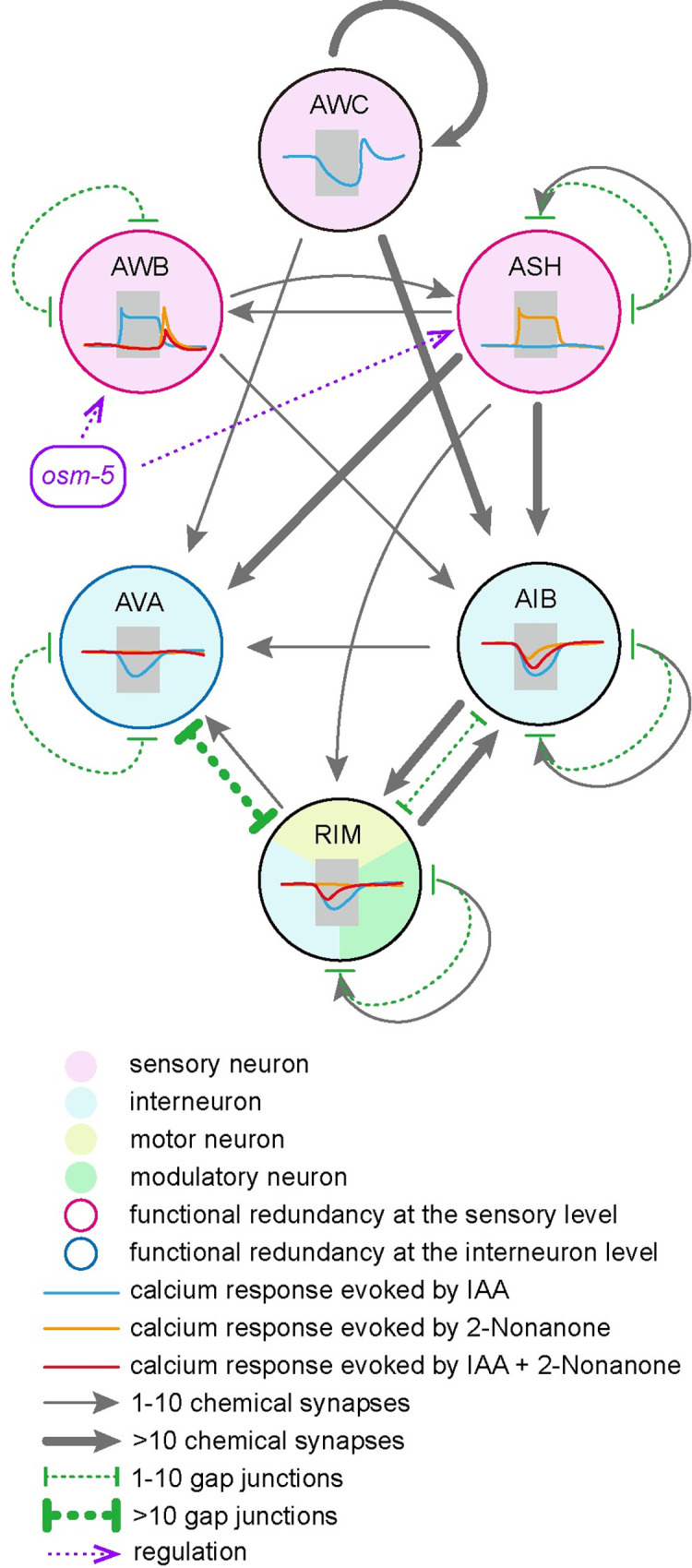
A model for redundant circuits underlying olfactory integration. We designed a new behavior paradigm to study a form of olfactory integration of repellent 2-nonanone and attractant IAA, which signal danger and food, respectively, in *C*. *elegans*. Our results suggest that AWB and ASH sensory neurons initiate redundant circuits to regulate integrated response to the two odorants. The signals generated by 2-nonanone and IAA are integrated at the levels of sensory neurons and downstream interneurons, including AVA, to weaken the signal and attraction evoked by IAA. Together, we propose that functional redundancy of neuronal circuits ensures the avoidance behavior when 2-nonanone, a repulsive odorant signaling danger, is present simultaneously with attractive odorants, such as IAA. Note: the synapses and gap junctions are illustrated based on the outputs from http://www.nemanode.com [52].

In this study, we developed a new behavior paradigm to study olfactory integration using a mixture of two odorants of opposing valences, the repulsive odorant 2-nonanone and the attractive odorant IAA that respectively represent danger and reward. Wild-type animals show a near complete suppressing effect of 2-nonanone on IAA. Mutations in *osm-5*, *osm-1* and likely in *dyf-7* that are known to regulate the development and function of cilia and sensory neurons show an impaired integrated response when 2-nonanone is paired with IAA. By analyzing the function of *osm-5*, we find that OSM-5 acts in either AWB or ASH, two pairs of sensory neurons that detect repellents and mediate avoidance response, to regulate olfactory integration. AWB and downstream interneurons AIB, AVA and RIM process the sensory responses to 2-nonanone and IAA to generate a neural circuit for olfactory integration (Fig 8).

### The sensory cilia are important for olfactory integration

The cilia of *C*. *elegans* sensory neurons play an important role in sensing external cues. As major sensory machineries, the cilia contain sensory receptors, signaling proteins and display specialized morphologies [46]. Thus, an intact cilium is important for sensorimotor behavior of worms, including olfaction. Previous studies have shown that mutations disrupting cilia structure, such as those in *bbs-7* or *bbs-8* or *odr-3*, reduce chemotaxis to odorants, including IAA [47,48]. By analyzing about 20,000 mutants generated from random mutagenesis, we found that mutations that inactivated genes involved in ciliogenesis or development of sensory neurons disrupted the integrated response to pairing of 2-nonanone with the attractive odorant IAA. We further showed that inactivating a ciliogenesis gene, *osm-5*, weakened the neuronal responses of AWB to IAA and 2-nonanone either in isolation or as a mixture. The *osm-5* mutation also altered the responses of downstream interneurons, such as AVA and AIB, to the mixture of the two odorants. These findings suggest a role of cilia in regulating odor integration.

### Redundant circuits are used for olfactory integration

Instinctive behaviors, such as feeding and avoiding dangers, are essential for survival. Thus, the underlying neural circuits need to be able to generate robust behavioral outputs. This demand is partly met through redundancy of the circuits. For example, subsets of the Agouti related protein-expressing neurons in the brain form redundant circuits, each of which is sufficient to regulate feeding behavior in mice [49]. However, anatomically redundant neurons do not necessarily lead to functional redundancy. For example, the sister mitral cells innervating the same glomerulus in the mammalian olfactory bulb generate different spike patterns in response to the same sensory input [50]. Thus, functional redundancy cannot be predicted solely based on anatomical properties. Previous studies show that both AWB and ASH sensory neurons regulate the avoidance of 2-nonanone, but in different ways. AWB neurons respond to a decrease in 2-nonanone concentration with increased intracellular calcium transients and integrate the signal over time. In contrast, ASH respond to an increase in 2-nonanone concentration to trigger reversals [23]. Thus, it is likely that AWB and ASH neurons signal to separate downstream circuits to process information of 2-nonanone and generate behavioral outputs. In our study, we observed that restoring the expression of *osm-5* in either AWB or ASH neurons almost fully rescued the olfactory effect of 2-nonanone on IAA, suggesting their redundant function in olfactory integration (Fig 8). Because *C*. *elegans* showed instinctive avoidance to 2-nonanone, this circuit redundancy may be important to generate robust avoidance of dangers in the presence of food signals in order to benefit survival.

### AWB, AVA, AIB and RIM neurons contribute to cellular basis of olfactory integration

AWB neurons can respond to both 2-nonanone and IAA cues, consistent with the possibility that AWB integrate these two signals [23,45]. Based on our observation, GCaMP signal of AWB evoked by the mixture of 2-nonanone and IAA resembles that evoked by 2-nonanone alone, not that evoked by IAA. These results suggest that AWB response to 2-nonanone overwrites its response to IAA and suggest integration at the sensory level. The *osm-5* mutants show partially reduced calcium responses to IAA or 2-nonanone, and are defective in IAA chemotaxis and 2-nonanone avoidance, indicating a role of OSM-5 in odorant sensation, consistent with the requirement of OSM-5 for cilia morphology [51]. Expressing *osm-5* in AWB fully rescues the calcium response evoked by IAA or by the mixture of 2-nonanone and IAA, suggesting that the full response of AWB to IAA and to the odorant mixture depends on the integrity of cilia. Interestingly, AWB expression of *osm-5* only rescues the latency, but not the amplitude, of the calcium response to 2-nonanone, and rescues the avoidance of 2-nonanone in behavior. This results suggest that the amplitude of 2-nonanone-evoked calcium response in AWB is not required to induce avoidance behavior. Our results also suggest a role of OSM-5 in regulating the integration of these 2 signals, as AWB neurons of *osm-5* mutants show a reduced and delayed OFF response to the mixture, which can be fully rescued by restoring OSM-5 in AWB. Overall, our data support that AWB is one of the neurons regulating olfactory integration, the temporal control of the OFF response by OSM-5 is critical for generating robust avoidance to either 2-nonanone or the mixture.

Our findings showing the responses of AVA, AIB and RIM interneurons to the mixture of IAA and 2-nonanone provides part of the mechanism for how the deeper layers of the neuronal network regulate integration. The contributions of these three pairs of interneurons to olfactory integration are different. In AVA neurons, exposure to 2-nonanone blocks IAA signals, although AVA seems to be quiet in response to the stimulation of 2-nonanone. These results suggest that the sensory response evoked by 2-nonanone is likely to silence AVA neurons, which blocks its response to IAA and, thus, contributes to olfactory integration. In AIB neurons, the calcium responses evoked by IAA or 2-nonanone or the mixture are all suppressive and regulated by *osm-5*, suggesting that AIB have dynamic roles in responding to the signals generated by these two olfactory stimuli in an *osm-5-*dependent manner. RIM neurons are generally processing IAA-related signals, which is independent of *osm-5*. We propose that AIB and RIM relay and/or process odorant signals for integration to take place somewhere else in the circuit, such as interneurons AVA which receive synaptic inputs from both AIB and RIM. Inactivating these neurons or blocking their synaptic outputs does not disrupt the integrated behavioral response, possibly due to redundant function of these and several other interneurons, such as AIA and AIY [52] that are synaptically connected with both IAA-sensing and 2-nonanone-sensing sensory neurons and downstream motor circuit [14]. Overall, we propose that IAA signals are blocked by 2-nonanone signals in both the sensory neurons and interneurons and that olfactory integration is achieved through reducing IAA-related responses at multiple steps of signal transmission.

In addition, the calcium response of AVA neurons to 2-nonanone reveals an intriguing pattern. We showed that 2-nonanone did not evoke an obvious calcium response in AVA, while it had the tendency to abolish IAA signals. This observation is surprising, as no calcium response usually suggests that a neuron is not responsive to the stimulus. Our data suggest that 2-nonanone evoked signaling silences AVA to abolish IAA-induced suppression. ASH and AWB neurons are the main sensory neurons responding to 2-nonanone. ASH neurons have 36 synapses onto AVA neurons, while AWB neurons have none [52]. Because ASH neurons are glutamatergic [53], it will be interesting to examine glutamatergic signaling between ASH and AVA in order to understand the response of AVA to 2-nonanone.

### Olfactory integration is a context-dependent response to odorants

While the molecular and cellular mechanisms underlying olfactory sensation have been well studied using individual chemicals, the olfactory stimuli that an animal encounters in its daily life are often mixtures of different odorant chemicals. A previous study in *C*. *elegans* shows that an arthropod repellant DEET masks chemotaxis toward several odorants, including attractive odorants IAA, butanone, and diacetyl [13]. In *Drosophila*, it shows that mixing an aversive odorant with an attractive odorant can significantly reduce the behavioral attraction to the attractive odorant. The context-dependent response to the attractant is regulated by lateral inhibition to the glomerulus that responds to the attractant [54]. In mice, different ligands for the same trace amine-associated receptor can elicit different behaviors, likely due to different additional receptors activated by each of these ligands [3]. These context-dependent effects on behavioral response to odorants provide mechanisms through which the nervous system processes and integrates complex olfactory information. Here, we show that the presence of the repulsive odorant 2-nonanone completely suppresses the attraction of IAA in behavior and alters the responses of AWB sensory neurons and downstream interneurons evoked by IAA. These results show that when 2-nonanone, which indicates danger, is present as a context, both the behavioral and neuronal response to IAA are modulated. It is conceivable that these context-dependent regulations of olfactory response were built into the connectivity and function of the nervous system to ensure the avoidance of danger-associated signals.

## Materials and methods

### Strains and transgenes

*C*. *elegans* strains were maintained under standard conditions at 20°C [55]. Hermaphrodites were used in the study. The strains that were used include: N2, ZC2925 *dyf-7(yx49)* X, ZC2926 *osm-1(yx50)* X, ZC2927 *osm-5(yx51)* X, PR813 *osm-5(p813)* X, ZC2858 *osm-5(p813)* X; *yxEx1475[osm-5p*::*osm-5*, *unc-122p*::*GFP]*, ZC2868 *osm-5(yx51)* X; *yxEx1483[osm-5p*::*osm-5*, *unc-122p*::*GFP]*, ZC2880 *osm-5(p813)* X; *yxEx1489[str-1p*::*osm-5*, *unc-122p*::*GFP]*, ZC2910 *osm-5(p813)* X; *yxEx1513[str-1p*::*osm-5*, *unc-122p*::*GFP]*, ZC2911 *osm-5(p813)* X; *yxEx1514[sra-6p*::*osm-5*, *unc-122p*::*GFP]*, ZC2912 *osm-5(p813)* X; *yxEx1515[sra-6p*::*osm-5*, *unc-122p*::*GFP]*, ZC2919 *osm-1(yx50)* X; *yxEx1522[Fosmid WRM0638dE09*, *unc-122p*::*GFP]*, ZC2904 *yxEx1507[str-1p*::*GCaMP6s*, *unc-122p*::*DsRed]*, ZC2921 *osm-5(p813)* X; *yxEx1507[str-1p*::*GCaMP6s*, *unc-122p*::*DsRed]*, ZC2905 *yxEx1508[sra-6p*::*GCaMP6s*, *unc-122p*::*DsRed]*, ZC2923 *osm-5(p813)* X; *yxEx1508[sra-6p*::*GCaMP6s*, *unc-122p*::*DsRed]*, ZC3170 *osm-5(p813)* X; *yxEx1643[str-1p*::*GCaMP6s*, *str-1p*::*osm-5*, *unc-122p*::*GFP]*, ZC3148 *osm-5(p813)* X; *yxEx1508[sra-6p*::*GCaMP6s*, *unc-122p*::*DsRed]*; *yxEx1514[sra-6p*::*osm-5*,*unc-122p*::*GFP]*, CX14996 *kyEx4965 [inx-1p*::*GCaMP3*, *tdc-1p*::*GCaMP3*, *rig-3p*::*GCaMP3*, *unc-122p*::*dsRed]*, ZC2965 *osm-5(p813)* X; *kyEx4965 [inx-1p*::*GCaMP3*, *tdc-1p*::*GCaMP3*, *rig-3p*::*GCaMP3*, *unc-122p*::*dsRed]* [37], PSC67 *scnEx46[inx-1p(AIBp)*::*twk-18gf-sl2-TagRFP*, *unc-122p*::*GFP*, *pUC19]*, PSC68 *scnEx47[inx-1p(AIBp)*::*twk-18gf-sl2-TagRFP*, *unc-122p*::*GFP*, *pUC19]*, PSC76 *scnEx55[inx-1p(AIBp)*::*twk-18gf-sl2-TagRFP*, *unc-122p*::*GFP*, *pUC19]*, PSC82 *scnEx56[nmr-1p*::*twk-18gf-sl2-TagRFP*, *unc-122p*::*GFP*, *pUC19]*, PSC90 *scnEx64[nmr-1p*::*TeTx-sl2-TagRFP*, *unc-122p*::*GFP*, *pUC19]*, JN1713 *peIs1713[sra-6p*::*mCasp-1*, *unc-122p*::*mCherry]*[45], JN1715 *peIs1715[str-1p*::*mCasp-1*, *unc-122p*::*GFP]* [45], PY7502 *oyIs85[ceh-36p*::*TU#813*, *ceh-36p*::*TU#814*, *srtx-1p*::*GFP*, *unc-122p*::*DsRed]* [56].

To generate transgenic animals, fosmid WRM0638dE09 (Wellcome Trust Sanger Institute) was injected into *yx50* at 5 ng/μL. The 242 bp 5’ upstream sequence of *osm-5* was generated by PCR (Primers: 5’-tttattgttttgaaattgaaagactcg-3’ and 5’-taagaaaagtgttctcagaagaaatagag-3’) and cloned into PCR8 to generate the entry clone (pCR8-*osm-5p*). The *osm-5* cDNA sequence and GCaMP6s coding sequence were used to generate the destination vector pDEST-*osm-5* and pDEST-GCaMP6s by Gateway vector conversion kit. To generate *osm-5p*::*osm-5*, Gateway LR reaction was performed between pDEST-*osm-5* and PCR8-*osm-5p*. *osm-5p*::*osm-5* was injected into *yx51* or *p813* at 36 ng/μL. The 4700 bp 5’ upstream sequence of *str-1* [4] was cloned into pCR8 to generate the entry clone (PCR8-*str-1p*). The 3269 bp 5’ upstream sequence of *sra-6* [34] was cloned into pCR8 to generate the entry clone (PCR8-*sra-6p*). To generate plasmids *str-1p*::*osm-5* (injected at 37 ng/μL or 5 ng/μL), *sra-6p*::*osm-5* (injected at 5 ng/μL), *str-1p*::*GCaMP6s* (injected at 25 ng/μL), *sra-6p*::*GCaMP6s* (injected at 34 ng/μL), Gateway LR reactions were performed between above mentioned corresponding destination vectors and entry vectors. The plasmids *inx-1p(AIBp)*::*twk-18gf-sl2-RFP* (injected at 34 ng/μL), *nmr-1p(AVA/RIMp)*::*twk-18gf-sl2-RFP* (injected at 31 ng/μL), *nmr-1p(AVA/RIMp)*::*TeTx-sl2-RFP* (injected at 49 or 36 ng/μL) were constructed by Gibson assembly method following the manufacturer’s protocol using the fragments generated by the primer sets described below. The *inx-1* and *nmr-1* promoters were defined according to previous studies [57–59]. Microinjection (about 100 ng/μL total DNA concentration with PUC19 added when needed) was performed as described previously [60] with either *unc-122p*::*GFP* or *unc-122p*::*DsRed* at 30 ng/μL as a co-injection marker.

Gibson assembly fragments were generated by PCR. The plasmid *inx-1p(AIBp)*::*twk-18gf-sl2-RFP* was assembled using 3 fragments: using primers ypr333/ypr255 and template plasmid *myo-3p*::*CHR2*::*sl2*::*RFP* for fragment 1; using primers ypr753/ypr750 and genomic DNA of wild-type worms for fragment 2; using primers ypr751/ypr752 and template plasmid *twk-18(gf)*::*mCherry* for fragment 3. The *nmr-1p(AVA/RIMp)*::*twk-18gf-sl2-RFP* was assembled using 3 fragments: using primers ypr751/ypr779, ypr778/ypr333 and template plasmid *inx-1p(AIBp)*::*twk-18gf-sl2-RFP* for fragment 1 and 2; using primers ypr776/ypr777 and genomic DNA of wild-type worms for fragment 3. The *nmr-1p(AVA/RIMp)*::*TeTx-sl2-RFP* was assembled by 2 fragments; using primers ypr850/ypr333 and template plasmid *gpa-11p*::*TeTx-sl2-RFP* for fragment 1; using primers ypr776/ypr851 and genomic DNA of wild-type worms for fragment 2. Primers related to Gibson assembly are ypr255, ttgccatgttgttaccttgtat; ypr333, aagcttggcgtaatcatggtc; ypr750 gcgcaacaatcgccatggcggacaagaactgcaatg; ypr751, atggcgattgttgcgcaag; ypr752, ggtaacaacatggcaactagatgtcatgctctagatagtc; ypr753 tgattacgccaagcttattaaacacgcgggaaatt; ypr776, tgattacgccaagcttgatgattatggaaccaaactcag; ypr777, gcgcaacaatcgccatatctgtaacaaaactaaagtttgtcg; ypr778, caaccacacccagggcatccccgacttctttaagcag ypr779, ctgcttaaagaagtcggggatgccctgggtgtggttg; ypr850, atgccgatcaccatcaacaac; ypr851, tgatggtgatcggcatatctgtaacaaaactaaagtttgtcg

### Behavior assay

One-day old adult animals (more than 50 worms for each assay) were washed 4 times by M9 buffer (3g/L KH_2_PO_4_, 6g/L Na_2_HPO_4_, 5g/L NaCl, 0.12g/L MgSO_4_), and then placed at the center (marked with a “X” in Fig 1A) of a 10 cm NGM-agar plate (3g/L NaCl, 1.6% agar, 25mM KPO_4_ pH6.0 buffer, 1mM MgSO_4,_ 1mM CaCl_2_, 5mg/L Cholesterol). The odorants were placed 3.33 cm away from the center of the 10 cm NGM-agar plate and the control (NGM buffer) was on the opposite side. 1 μL of 1 mol/L sodium azide was placed beside the odorants and on the other side of the plate equidistant from the center to immobilize the worms that reach the odorants or the control. The extra M9 buffer was then removed by Kimwipes. The plate was sealed by Parafilm and placed upside-down on the bench for 1 hour. The numbers of worms in all three areas, as shown in Fig 1A, were counted manually, and the chemotaxis index (CI) and the integration index were calculated according to the equations in Fig 1A. The worms stayed in the middle area were the ones made no choice. The worms moved to the area with odor(s) were the ones that made a correct choice to IAA, and a wrong choice to 2-nonanone or IAA + 2-nonanone. The worms stayed in the area furthest away from the odor(s) were the ones that made a wrong choice to IAA, and a correct choice to 2-nonanone or IAA + 2-nonanone. We defined the incorrect choice as the sum of the wrong choice and no choice. If transgenic strains were used, animals with or without the transgenes were counted separately, based on the presence or absence of the co-injection marker. Statistical analyses were performed using GraphPad Prism.

### Mutagenesis, screen, and mutant identification

To generate mutants for forward genetic screen, L4-stage wild-type hermaphrodites (P0) were treated with 0.5% ethanemethylsulfonate (EMS diluted with M9 buffer) for 4 hours and washed with M9 buffer for four times. After recovering on a regular cultivation plate for overnight, 200 EMS-treated P0 worms were transferred to 50 fresh cultivation plates (4 worms/plate) to reproduce and removed after around 50 eggs (F1s) were found on each plate. F1 worms were removed after 200 ~ 500 eggs (F2) were found on each plate and F2 worms were tested for olfactory integration using 2-nonanone and IAA. The F2 worms stayed on the side of the odorant mixture were collected and cultivated into individual F2 clones for future analysis. Mutants that were morphologically defective, or severely uncoordinated in locomotion, or retarded in development, or strongly defective in single odorant chemotaxis were eliminated. Based on these criteria, 3 F2 clones, *yx49*, *yx50* and *yx51*, were identified as mutants for olfactory integration.

All three mutants were outcrossed three to four times with a wild-type genetic background and sequenced by Illumina Hi-Seq 2500 (single-read, 50-basepair read length). Sequencing reads were aligned to the WS260 reference genome and analyzed by MiModD (http://mimodd.readthedocs.io/en/latest/). The function of candidate genes, suggested by MiModD, in olfactory integration was tested by using the existing mutant allele(s) for the genes to see if they phenocopied the mutants identified from EMS-mutagenesis. If no mutant was available for a gene of interest, a fosmid containing the gene of interest was tested for its rescuing effect.

### Calcium imaging

Calcium imaging was performed using a microfluidic device essentially as we previously published [61–63]. Briefly, fresh solutions were prepared before each recording session by dissolving the tested chemical(s) in nematode growth medium buffer (3 g/L NaCl, 1 mM CaCl_2_, 1 mM MgSO_4_, 25 mM potassium phosphate buffer pH6.0) to the indicated concentration(s). We used 10^5^ x diluted 2-nonanone for imaging, which is about 57.6 uM (142.2g/mol, density 0.832g/mL, 1uL pure 2-nonanone diluted by 100mL NGM buffer), comparable to the maximal concentration of 2-nonanone (~ 20 uM) measured on the assay plates in chemotaxis in a previous study [23]. We used 10^4^ x diluted IAA because it was commonly used to analyze neuronal responses to IAA in previous studies [27,45]. Fluorescence time-lapse imaging was recorded using a Nikon Eclipse Ti-U inverted microscope with a 40X oil immersion objective and a Yokogawa CSU-X1 scanner unit and a Photometrics CoolSnap EZ camera at 5 frames per second. The GCaMP signal from the soma of AWB or ASH was measured using Fiji. The change in the fluorescence intensity (ΔF) for each frame was the difference between its fluorescence intensity and the average intensity over the 10-second recording before the stimulus onset (F_base_): ΔF = F—F_base_. To analyze the response evoked by the onset of the stimulus for each genotype (ON response), the average ΔF/F_base_ % during the 5-second (for AVA, AIB and RIM) or 10-second window (for AWB and ASH) after onset or 2^nd^ 10-second window after onset (for AVA) minus average ΔF/F_base_ % of the 10-second window before onset was quantified. To analyze the response evoked by the removal of the stimulus (OFF response), the average ΔF/F_base_ % of the 5-second window (for AVA, AIB and RIM) or 10-second window (for AWB and ASH) after removal minus the average ΔF/F_base_ % of the 10-second window before removal was quantified. The latency for the OFF response was defined as the time needed for the calcium signal to reach the mean of 10-second window before removal + 3 x SD (standard deviation). If the calcium signal did not reach mean + 3 x SD by the end of recording, the latency was defined as 40 seconds. The statistical methods are shown in the figure legends.

### Dye filling assay

Dye filling were performed according to the protocol presented at wormatlas.org. Briefly, DiO stock solution (2 mg/mL) was diluted 200 times with M9 buffer immediately before use. Worms were washed 3 times and kept in a drop of M9 on an empty 6 cm plate. Then, 30–50 worms were transferred by a worm pick to an Eppendorf tube with 1 mL M9 buffer containing diluted DiO. This tube was kept from light for 2 hours at room temperature. After staining, the worms were transferred to a fresh plate containing an OP50 lawn and kept at room temperature for 1 hour. The worms were then immobilized by 20 mM sodium azide and put on an agar pad for fluorescence imaging.

## Supporting information

S1 Fig Expressing *osm-5* rescues the defects of *osm-5(yx51)* mutants. **(A—C)** Expressing a wild-type *osm-5* cDNA using an *osm-5* promoter rescues the defects of *osm-5(yx51)* mutants in chemotaxis to IAA and 2-nonanone (Nona) (**A**) and olfactory integration (**A, B**), as well as behavioral choices during the assays (**C**). For all, box plots indicate median, the first and the third quartile, and the minimal and maximal values. The numbers of assays are shown in the parentheses, which are highlighted in orange if the data are not normally distributed. Two tailed unpaired *t*-test (if data are normally distributed) or two tailed Mann-Whitney test (if data are not normally distributed) is used to compare transgenic animals and their non-transgenic siblings tested in parallel. **** *p* < 0.0001, *** *p* < 0.001, ** *p* < 0.01, * *p* < 0.05.
(TIF)

S2 Fig Mutants defective in olfactory integration are defective in DiO staining. Representative images of wild type and olfactory integration mutants after dye filling of DiO. Several neurons in wild type uptake DiO from the environment and generate fluorescent signals. In contrast, *yx51*, *yx50* and *yx49* mutants do not show a dye fill signal. The head of each worm is shown and dashed lines outline the worms.
(TIF)

S3 Fig The results from additional transgenic lines generated independently from those in Fig 4 show that *osm-5* acts in AWB or ASH neurons to regulate chemotaxis and olfactory integration. **(A—C)** Expressing a wild-type *osm-5* cDNA in AWB rescues the defects of *osm-5(p813)* mutants in chemotaxis to 2-nonanone (Nona) (**A**) and olfactory integration (**A, B**), as well as behavioral choices during the assays (**C**). **(D—F)** Expressing a wild-type *osm-5* cDNA in ASH rescues the defects of *osm-5(p813)* mutants in chemotaxis to 2-nonanone (Nona) (**D**) and olfactory integration (**D, E**), as well as behavioral choices during the assays (**F**). For all, box plots indicate median, the first and the third quartile, and the minimal and maximal values. The numbers of assays are shown in the parentheses, which are highlighted in orange if the data are not normally distributed. Two tailed unpaired *t*-test (if data are normally distributed) or two tailed Mann-Whitney test (if data are not normally distributed) is used to compare transgenic animals and their non-transgenic siblings tested in parallel. **** *p* < 0.0001, *** *p* < 0.001, ** *p* < 0.01, * *p* < 0.05, ns, not significant.
(TIF)

S4 Fig Quantitation of the results shown in Fig 7. **(A—I)** Quantitation of the results shown in Fig 7A–7I, respectively. For all, horizontal bars in each graph are median with 95% confidence interval, individual data points are shown as dots. The numbers of assays are shown in the parentheses, which are highlighted in orange if the data are not normally distributed. Two tailed unpaired *t*-test (if data are normally distributed) or two tailed Mann-Whitney test (if data are not normally distributed). *** *p* < 0.001, ** *p* < 0.01, * *p* < 0.05, ns, not significant.
(TIF)

S5 Fig Inactivating AVA, AIB, RIM or blocking their synaptic outputs does not impair olfactory integration in behavior. **(A—C)** Expressing a *twk-18(gf)* cDNA in AIB using *inx-1* promoter slightly impairs the integrated behavioral response in line PSC67 (**A**), and does not impair the behavior in another 2 lines (**B, C**). **(D)** Expressing a *twk-18(gf)* cDNA in AVA and RIM using *nmr-1* promoter does not impair the integration behavior. **(E)** Expressing tetanus toxin (TeTx) in AVA and RIM using *nmr-1* promoter does not impair the integration behavior. In **A–E,** box plots indicate median, the first and the third quartile, and the minimal and maximal values; the numbers of assays are indicated in the parentheses, which are highlighted in orange if the data are not normally distributed. Two tailed unpaired *t*-test (if data are normally distributed) or two tailed Mann-Whitney test (if data are not normally distributed) is used to compare transgenic animals and their non-transgenic siblings tested in parallel. **** *p* < 0.0001, ** *p* < 0.01, * *p* < 0.05, ns, not significant.
(TIF)

S6 Fig The *osm-5(p813)* mutant animals are defective in several olfactory tasks. **(A, B)** The *osm-5(p813)* mutant animals are defective in olfactory integration of benzaldehyde with 2-nonanone. **(C—E)** The *osm-5(p813)* mutant animals are defective in high concentration-dependent repulsion of IAA, benzaldehyde and diacetyl. For all, box plots indicate median, the first and the third quartile, and the minimal and maximal values. The numbers of assays are shown in the parentheses, which are highlighted in orange if the data are not normally distributed. Two tailed unpaired *t*-test (if data are normally distributed) or two tailed Mann-Whitney test (if data are not normally distributed) is used to compare wild type and *osm-5* mutant animals. **** p < 0.0001, *** p < 0.001, ** p < 0.01, ns, not significant.
(TIF)
