# Supplementary figures and images for: Redundant neural circuits regulate olfactory integration
Source: PLoS Genet. 2022 Jan 31;18(1):e1010029. doi: 10.1371/journal.pgen.1010029 (PMC8830790; doi:10.1371/journal.pgen.1010029)

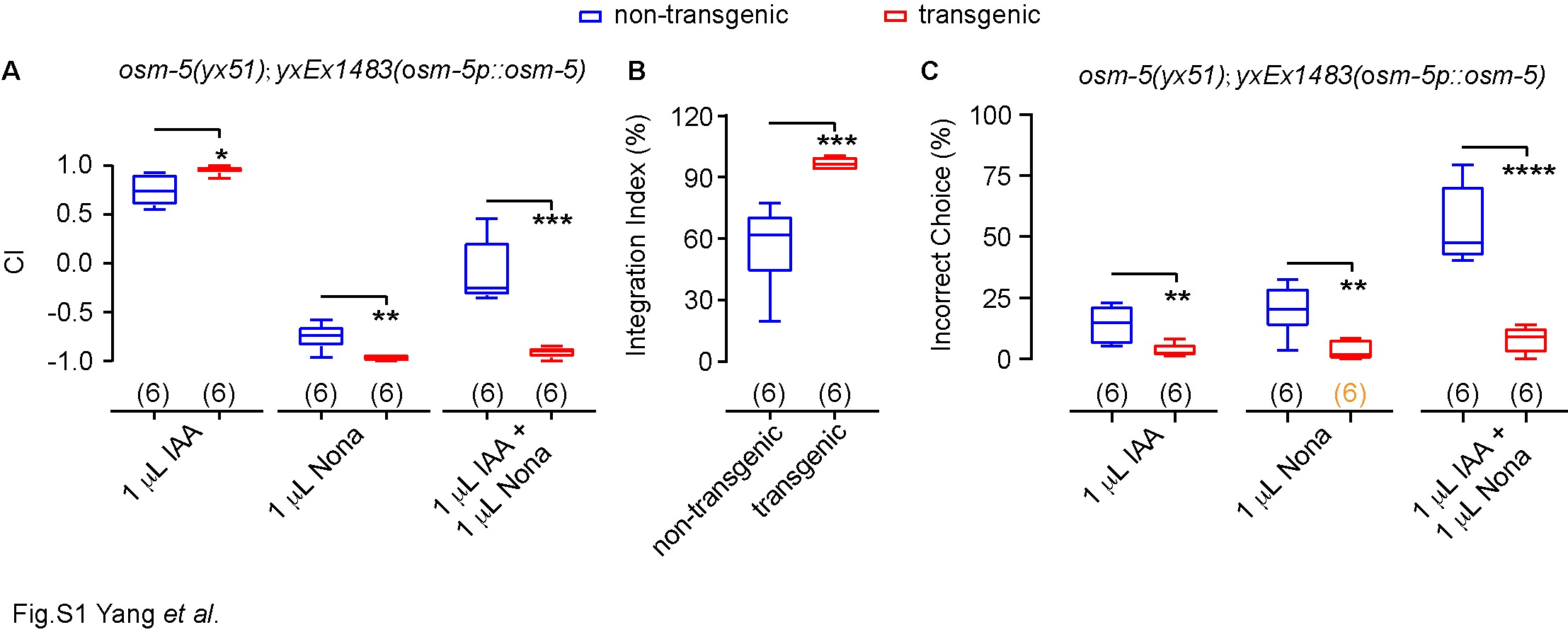

Supplement: S1 Fig — (A—C) Expressing a wild-type osm-5 cDNA using an osm-5 promoter rescues the defects of osm-5(yx51) mutants in chemotaxis to IAA and 2-nonanone (Nona) (A) and olfactory integration (A, B), as well as behavioral choices during the assays (C). For all, box plots indicate median, the first and the third quartile, and the minimal and maximal values. The numbers of assays are shown in the parentheses, which are highlighted in orange if the data are not normally distributed. Two tailed unpaired t-test (if data are normally distributed) or two tailed Mann-Whitney test (if data are not normally distributed) is used to compare transgenic animals and their non-transgenic siblings tested in parallel. **** p < 0.0001, *** p < 0.001, ** p < 0.01, * p < 0.05. (TIF) [file pgen.1010029.s001.tif]

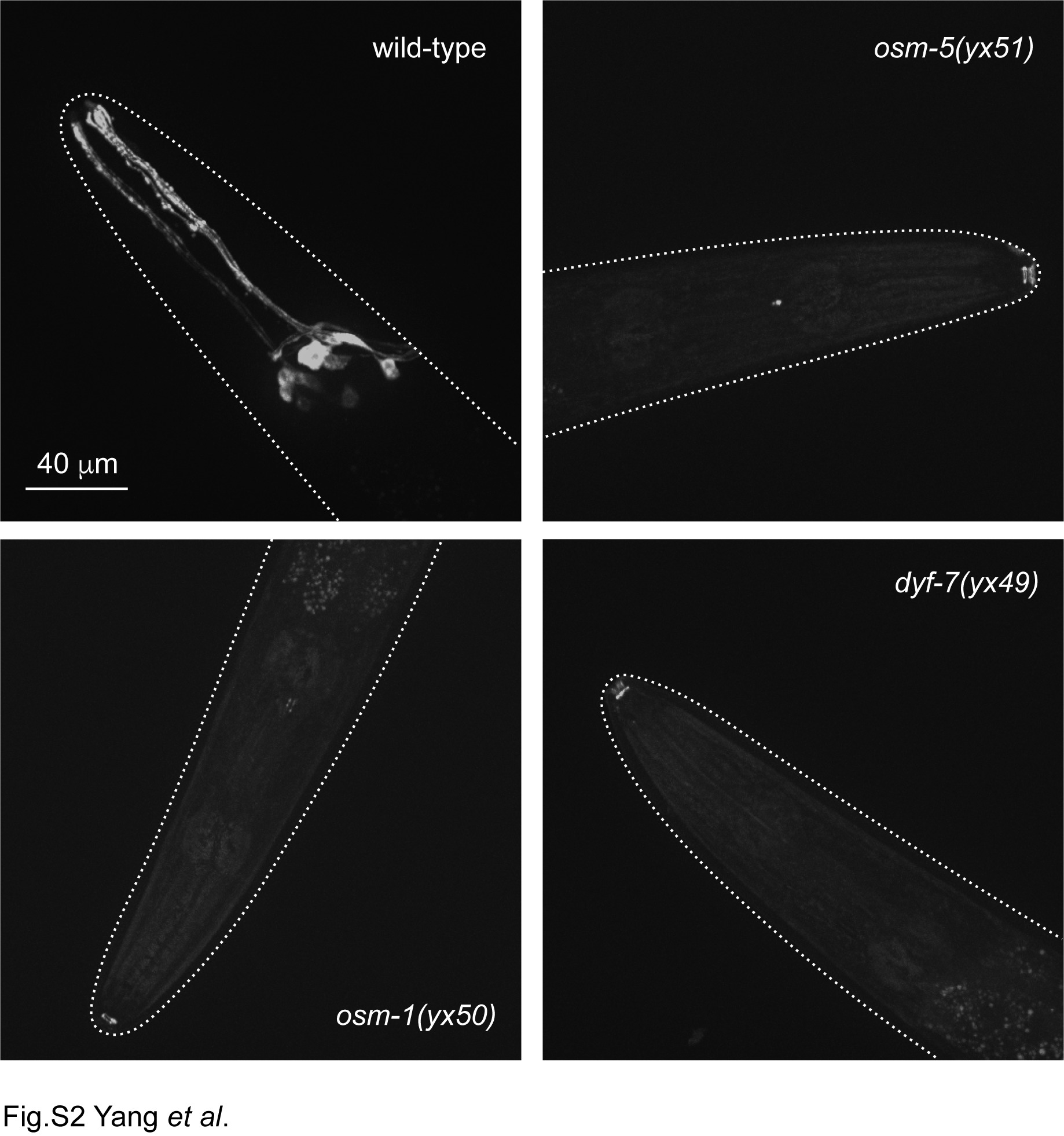

Supplement: S2 Fig — Representative images of wild type and olfactory integration mutants after dye filling of DiO. Several neurons in wild type uptake DiO from the environment and generate fluorescent signals. In contrast, yx51, yx50 and yx49 mutants do not show a dye fill signal. The head of each worm is shown and dashed lines outline the worms. (TIF) [file pgen.1010029.s002.tif]

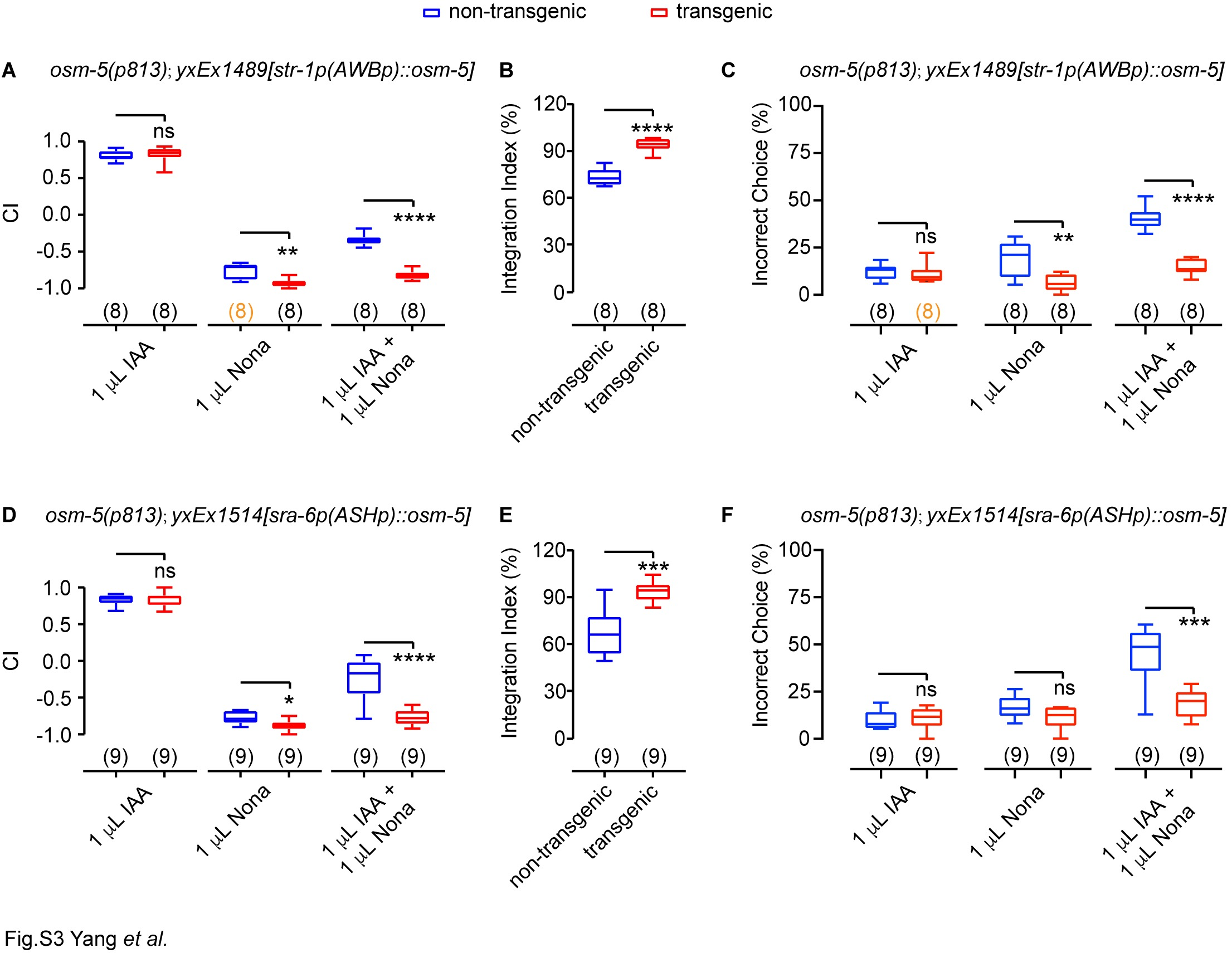

Supplement: S3 Fig — (A—C) Expressing a wild-type osm-5 cDNA in AWB rescues the defects of osm-5(p813) mutants in chemotaxis to 2-nonanone (Nona) (A) and olfactory integration (A, B), as well as behavioral choices during the assays (C). (D—F) Expressing a wild-type osm-5 cDNA in ASH rescues the defects of osm-5(p813) mutants in chemotaxis to 2-nonanone (Nona) (D) and olfactory integration (D, E), as well as behavioral choices during the assays (F). For all, box plots indicate median, the first and the third quartile, and the minimal and maximal values. The numbers of assays are shown in the parentheses, which are highlighted in orange if the data are not normally distributed. Two tailed unpaired t-test (if data are normally distributed) or two tailed Mann-Whitney test (if data are not normally distributed) is used to compare transgenic animals and their non-transgenic siblings tested in parallel. **** p < 0.0001, *** p < 0.001, ** p < 0.01, * p < 0.05, ns, not significant. (TIF) [file pgen.1010029.s003.tif]

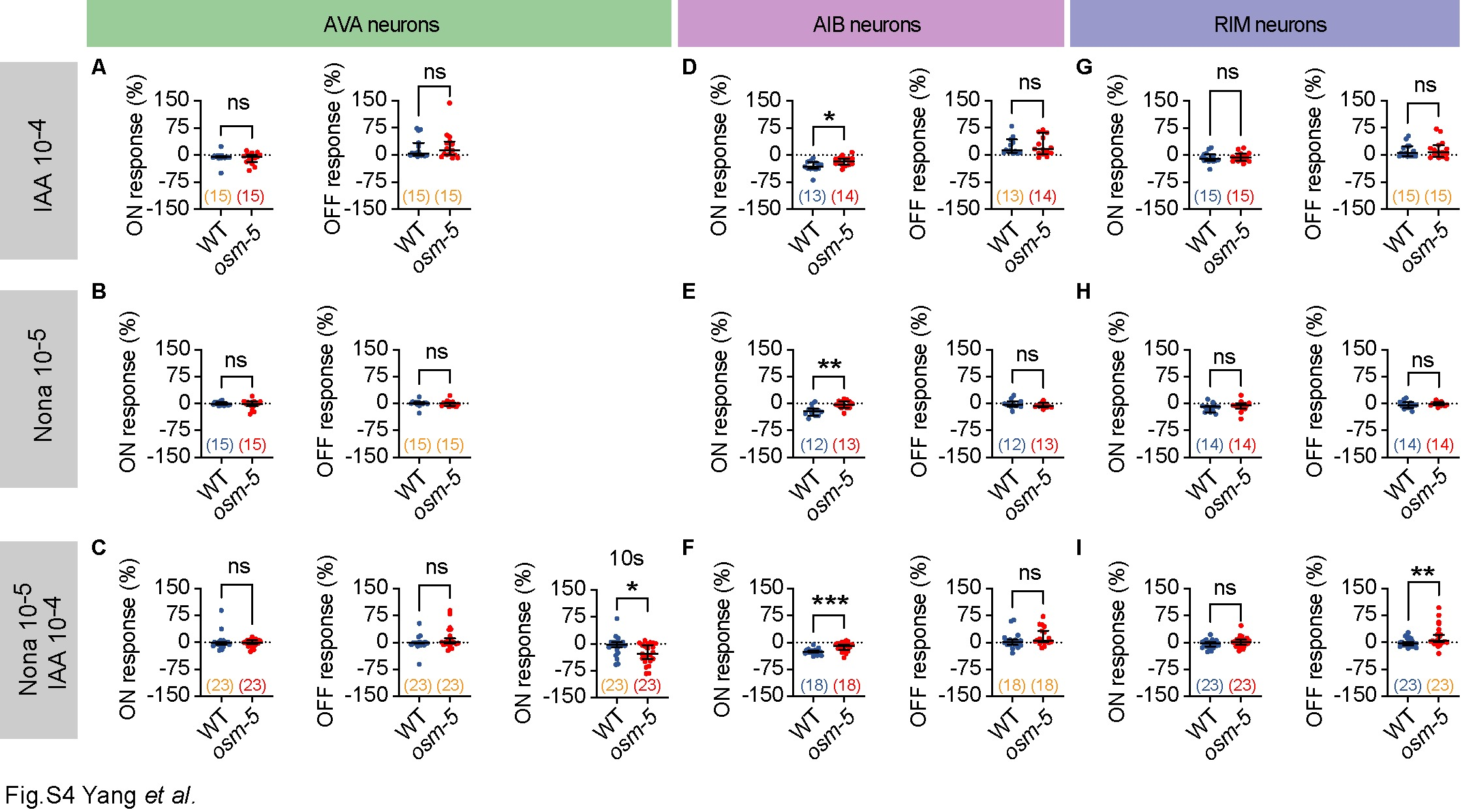

Supplement: S4 Fig — (A—I) Quantitation of the results shown in Fig 7A–7I, respectively. For all, horizontal bars in each graph are median with 95% confidence interval, individual data points are shown as dots. The numbers of assays are shown in the parentheses, which are highlighted in orange if the data are not normally distributed. Two tailed unpaired t-test (if data are normally distributed) or two tailed Mann-Whitney test (if data are not normally distributed). *** p < 0.001, ** p < 0.01, * p < 0.05, ns, not significant. (TIF) [file pgen.1010029.s004.tif]

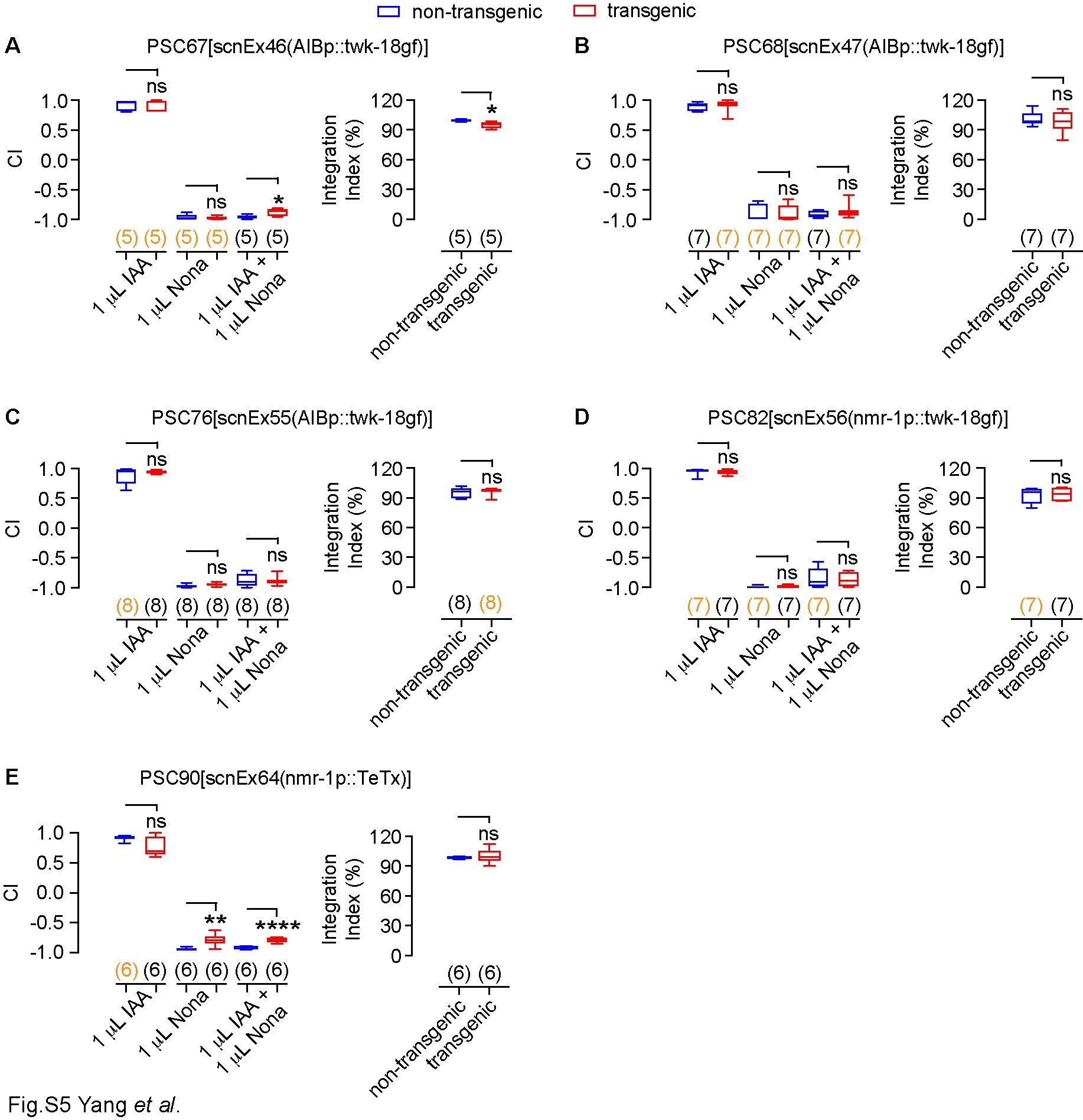

Supplement: S5 Fig — (A—C) Expressing a twk-18(gf) cDNA in AIB using inx-1 promoter slightly impairs the integrated behavioral response in line PSC67 (A), and does not impair the behavior in another 2 lines (B, C). (D) Expressing a twk-18(gf) cDNA in AVA and RIM using nmr-1 promoter does not impair the integration behavior. (E) Expressing tetanus toxin (TeTx) in AVA and RIM using nmr-1 promoter does not impair the integration behavior. In A–E, box plots indicate median, the first and the third quartile, and the minimal and maximal values; the numbers of assays are indicated in the parentheses, which are highlighted in orange if the data are not normally distributed. Two tailed unpaired t-test (if data are normally distributed) or two tailed Mann-Whitney test (if data are not normally distributed) is used to compare transgenic animals and their non-transgenic siblings tested in parallel. **** p < 0.0001, ** p < 0.01, * p < 0.05, ns, not significant. (TIF) [file pgen.1010029.s005.tif]

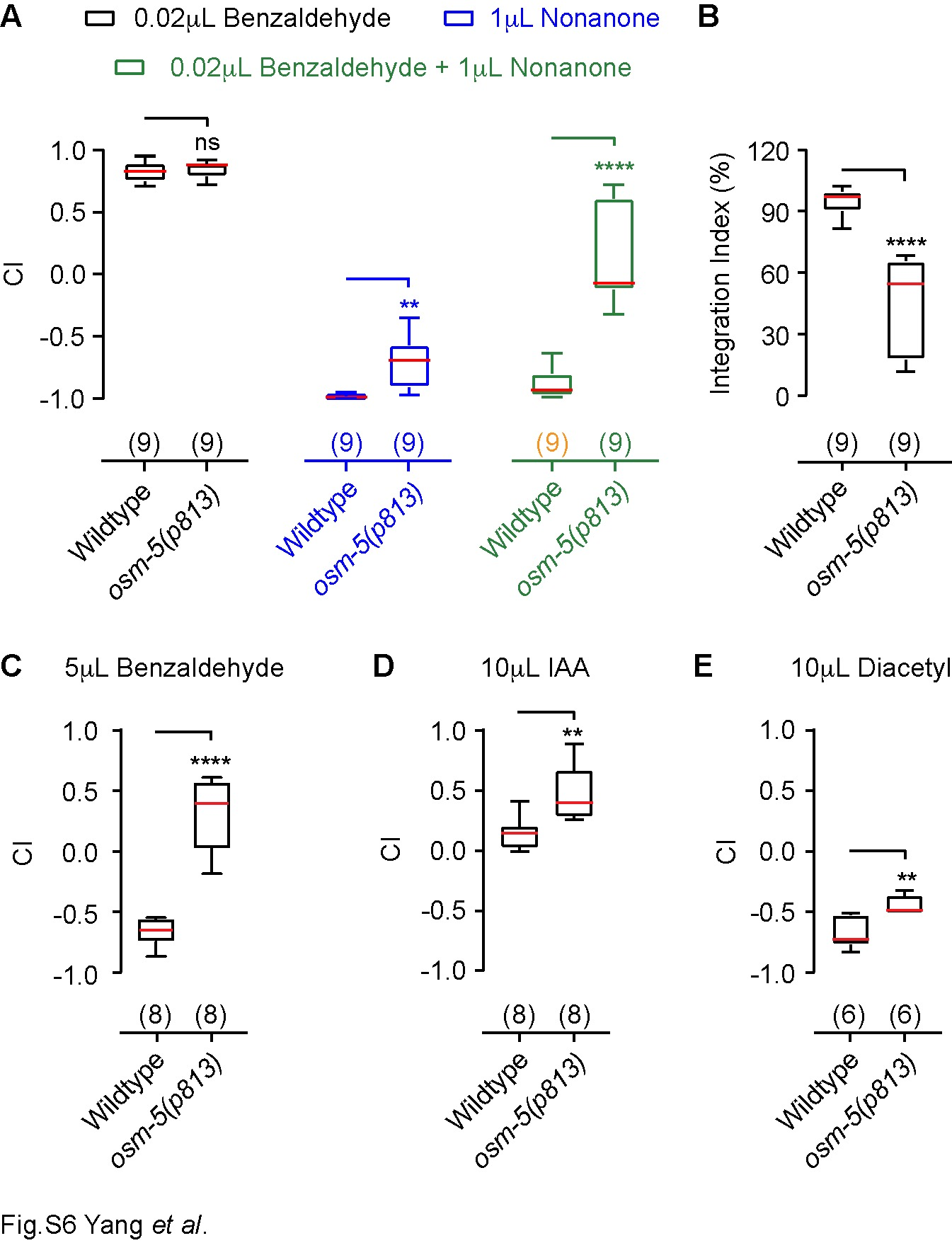

Supplement: S6 Fig — (A, B) The osm-5(p813) mutant animals are defective in olfactory integration of benzaldehyde with 2-nonanone. (C—E) The osm-5(p813) mutant animals are defective in high concentration-dependent repulsion of IAA, benzaldehyde and diacetyl. For all, box plots indicate median, the first and the third quartile, and the minimal and maximal values. The numbers of assays are shown in the parentheses, which are highlighted in orange if the data are not normally distributed. Two tailed unpaired t-test (if data are normally distributed) or two tailed Mann-Whitney test (if data are not normally distributed) is used to compare wild type and osm-5 mutant animals. **** p < 0.0001, *** p < 0.001, ** p < 0.01, ns, not significant. (TIF) [file pgen.1010029.s006.tif]
